# Engineering a short-chain dehydrogenase/reductase for the stereoselective production of (2*S*,3*R*,4*S*)-4-hydroxyisoleucine with three asymmetric centers

**DOI:** 10.1038/s41598-017-13978-w

**Published:** 2017-10-20

**Authors:** Xuan Shi, Takuya Miyakawa, Akira Nakamura, Feng Hou, Makoto Hibi, Jun Ogawa, Yeondae Kwon, Masaru Tanokura

**Affiliations:** 1 Laboratory of Basic Science on Healthy Longevity, Department of Applied Biological Chemistry, Graduate School of Agricultural and Life Sciences, The University of Tokyo, 1-1-1 Yayoi, Bunkyo-ku, Tokyo, 113-8657 Japan; 2 Industrial Microbiology, Graduate School of Agriculture, Kyoto University, Kitashirakawa-oiwakecho, Sakyo-ku, Kyoto, 606-8502 Japan; 3 Division of Applied Life Sciences, Graduate School of Agriculture, Kyoto University, Kitashirakawa-oiwakecho, Sakyo-ku, Kyoto, 606-8502 Japan

## Abstract

Fenugreek is a dietary supplement for anti-aging and human health. (2*S*,3*R*,4*S*)-4-hydroxyisoleucine (4-HIL), which is extracted from fenugreek seeds, is expected to be a promising orally active drug for diabetes and diabetic nephropathy because of its insulinotropic effect. Although several chemical synthesis methods of 4-HIL have been proposed, these methods require multistep reactions to control the stereochemistry of 4-HIL. In this study, we modified the key enzyme 4-HIL dehydrogenase (HILDH) to overcome the biggest limitation in commercial-scale production of 4-HIL. As a result, an effective one-step carbonyl reduction to produce (2*S*,3*R*,4*S*)-4-HIL was successfully accomplished with strict stereoselectivity (>99% de). Mass production of (2*S*,3*R*,4*S*)-4-HIL by our synthetic method could have a significant contribution to the prevention of diabetes, dyslipidemia, and Alzheimer’s disease. (120 words/200 words)

## Introduction

Fenugreek (*Trigonella foenum-graecum*) is one of the oldest and most promising medicinal herbs^1^. It has been in use for over 2,500 years, mainly in Asia, Africa and Latin America, and has been employed for numerous medicinal purposes including as an antibacterial, gastric stimulant and antidiabetic agent. In recent decades, the therapeutic benefits of fenugreek have been identified in animal studies as well as human trials, including antidiabetic^2–5^, anti-infective^6,7^, anti-inflammatory^8–11^, anticancer^12–15^, hypolipidemic^16–18^, hypocholesterolemic^2,3,5,19^, antioxidant^8,20–22^, cardioprotective^23–26^ and digestive stimulant activities^27^. 4-hydroxyisoleucine (4-HIL), which is extracted from fenugreek seeds, is a major compound that contributes to the physiological function of fenugreek. 4-HIL is a natural non-proteinogenic amino acid that can exist as eight stereoisomers because of its three chiral centers (Supplementary Fig. S1). Two diastereomers of 4-HIL were initially identified from fenugreek seeds^28^. The major one possesses a 2*S*,3*R*,4*S* configuration (up to 90% of total 4-HIL content of seeds) and the minor one has a 2*R*,3*R*,4*S* configuration^29^. (2*S*,3*R*,4*S*)-4-HIL has insulinotropic effect by directly affecting pancreatic B cells in rats and humans^30–35^. It has also been proven to possess insulin-sensitizing effects in liver and skeletal muscle^36^. A recent study showed that (2*S*,3*R*,4*S*)-4-HIL attenuates insulin resistance by decreasing tumor necrosis factor-α (TNF-α)^37^ and the activation of AMP-activated protein kinase (AMPK)^38^. Because of such insulinotropic effect and insulin sensitivity improvement, (2*S*,3*R*,4*S*)-4-HIL is expected to be a promising orally active drug for diabetes and diabetic nephropathy. In contrast to other antidiabetic agents, (2*S*,3*R*,4*S*)-4-HIL mediates insulin response and is strictly dependent on glucose concentration^30–36^. This unique property prevents undesirable side-effects such as hypoglycemia in the therapy of type 2 diabetes mellitus (T2DM). Meanwhile, as the number of elderly people increases, there will be a corresponding increase in the risk of Alzheimer’s disease (AD)^39–41^. It is hypothesized that many AD patients have comorbid T2DM^42^. The effects of insulin and glucose metabolism on the risk of developing dementia, especially AD, have been studied^43–45^. Therefore, increased insulin resistance is likely to lead not only to diabetes but also to dementia.

**Figure 1:**
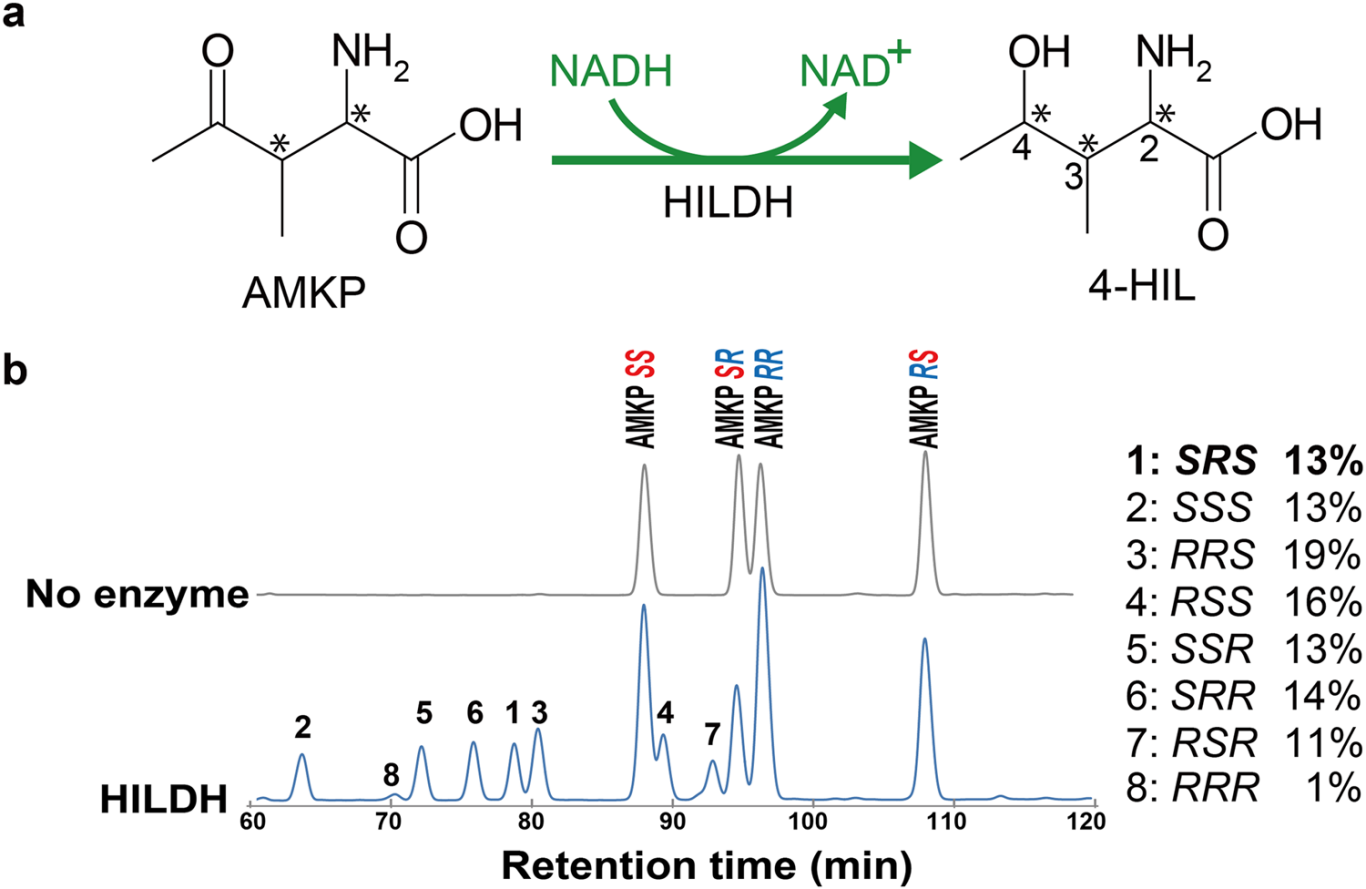
The enzymatic reaction of HILDH and 4-HIL stereoisomers production. (**a**) Schematic diagram of the enzymatic reaction of HILDH. HILDH reduces AMKP to 4-HIL in an NADH-dependent manner. Asymmetric centers are shown in asterisks. (**b**) HPLC chromatogram of 4-HIL stereoisomers produced (left) and their production ratios (right). The reaction mixtures were derivatized with GITC and detected by HPLC. Each peak label on the chromatogram (left) corresponds to each 4-HIL stereoisomer listed on the right. “No enzyme” indicates the reaction mixtures without HILDH.

Extraction of 4-HIL from fenugreek seeds is poorly suited to industrial scale-up because of low content (0.56 wt%)^35^. Several routes have been proposed for the synthesis of (2*S*,3*R*,4*S*)-4-HIL, such as traditional chemical methods or chemoenzymatic synthesis^46–48^. These methods require multistep reactions to control the stereochemistry of 4-HIL, with three chiral centers. Recently, 4-HIL dehydrogenase (HILDH) from *Bacillus thuringiensis* 2e2 was identified as a microbial enzyme that dehydrogenates (2*S*,3*R*,4*S*)-4-HIL into (2*S*,3*R*)-2-amino-3-methyl-4-ketopentanoic acid (AMKP) in the presence of NAD^+^^49^. HILDH belongs to the short-chain dehydrogenase/reductase (SDR) superfamily. Our further study found that HILDH could reversibly catalyze AMKP to produce 4-HIL. The racemic mixtures of AMKP that contains four stereoisomers could be reduced to eight possible stereoisomers of 4-HIL (Supplementary Fig. S1). HILDH reduces the racemic AMKP to (2*S*,3*R*,4*S*)-4-HIL and the other seven stereoisomers of 4-HIL in an NADH-dependent manner, suggesting that HILDH has nearly no stereoselectivity with AMKP (Fig. 1). The (2*S*,3*R*,4*S*)-4-HIL could be oxidized by HILDH in an NAD^+^-dependent manner and produces only one isomer, (2*S*,3*R*)-AMKP^49^. Therefore, the chirality of AMKP at the positions 2 and 3 is not converted during the reduction reaction by HILDH, which does not contradict the general reaction mechanism of SDR proteins. AMKP is known as a vitamin B12 antimetabolite and could be isolated as stereoisomeric mixtures from *Bacillus cereus* 439 fermentations^50^. The stereoisomeric mixtures of AMKP can be easily synthesized by condensation of 2-bromo-3-butanone and diethyl acetamidomalonate, followed by hydrolysis with 6 *N* HCl^50^. The stereoselectivity of HILDH is not strict enough for producing (2*S*,3*R*,4*S*)-4-HIL, as its production ratio is 13% among the eight 4-HIL stereoisomers, which are potentially produced by the reduction of the carbonyl group of the racemic AMPK, which itself occurs as four stereoisomers (Fig. 1b). Thus, protein engineering of HILDH is required to address the limited industrial uses of this enzyme. There are two main approaches for protein engineering: directed evolution and rational design. Because the directed evolution needs several rounds of evolution to be applied and numerous mutants to be screened, protein engineering is developing more and more from random approaches to rational design such as site-specific mutagenesis or structure-guided recombination^51^. In many case, changing of selectivity or activity of an enzyme usually targets directly the active site. Protein structure could be helped to understand the basis of reaction mechanism and provides appropriates ideas for point mutation. Thus, a structural basis for enhancing the stereoselectivity of (2*S*,3*R*,4*S*)-4-HIL could provide direction for the protein engineering of HILDH.

Reductases are currently the most promising kind of enzymes in producing chiral compounds due to their conciseness, low-cost and environment-friendly nature in reaction process. Therefore, optimizing reductases to improve their stereoselectivity also has been actively developed. In this study, we modified the key enzyme, HILDH by site-specific mutagenesis to improve its stereoselectivity toward (2*S*,3*R*,4*S*)-4-HIL. As a result, a double mutant of HILDH produces (2*S*,3*R*,4*S*)-4-HIL with strict stereoselectivity (>99% de). The native HILDH shows no stereoselectivity except for one stereoisomer. It reduces the racemic substrate with two asymmetric centers to produce seven stereoisomers among eight possible types. However, the engineered HILDH has strict stereoselectivity toward three asymmetric centers of 4-HIL. To the best of our knowledge, this study is the first to report engineering of an enzyme to improve stereoselectivity toward three asymmetric centers. In addition, an NADH regeneration system using formate dehydrogenase (FDH) was introduced to scale up the reaction. FDH has relatively low activity and is labile. To overcome such disadvantages, several FDH mutants have been developed to improve their catalytic properties and stability^52,53^. Besides, considering the ease of separating product carbon dioxide, NADH recycling coupled with FDH is economically viable in the industrial use of NADH-dependent HILDH. Mass production of this important amino acid using this synthetic method could lead to a significant contribution in the prevention of diabetes, dyslipidemia, and AD. In addition, it establishes the value of food for disease prevention beyond the conventional view that dietary supplements and herbs can only be taken for health maintenance.

## Results and Discussion

### Crystal structure of HILDH and 4-HIL-binding mode in the active site of HILDH

**Figure 2:**
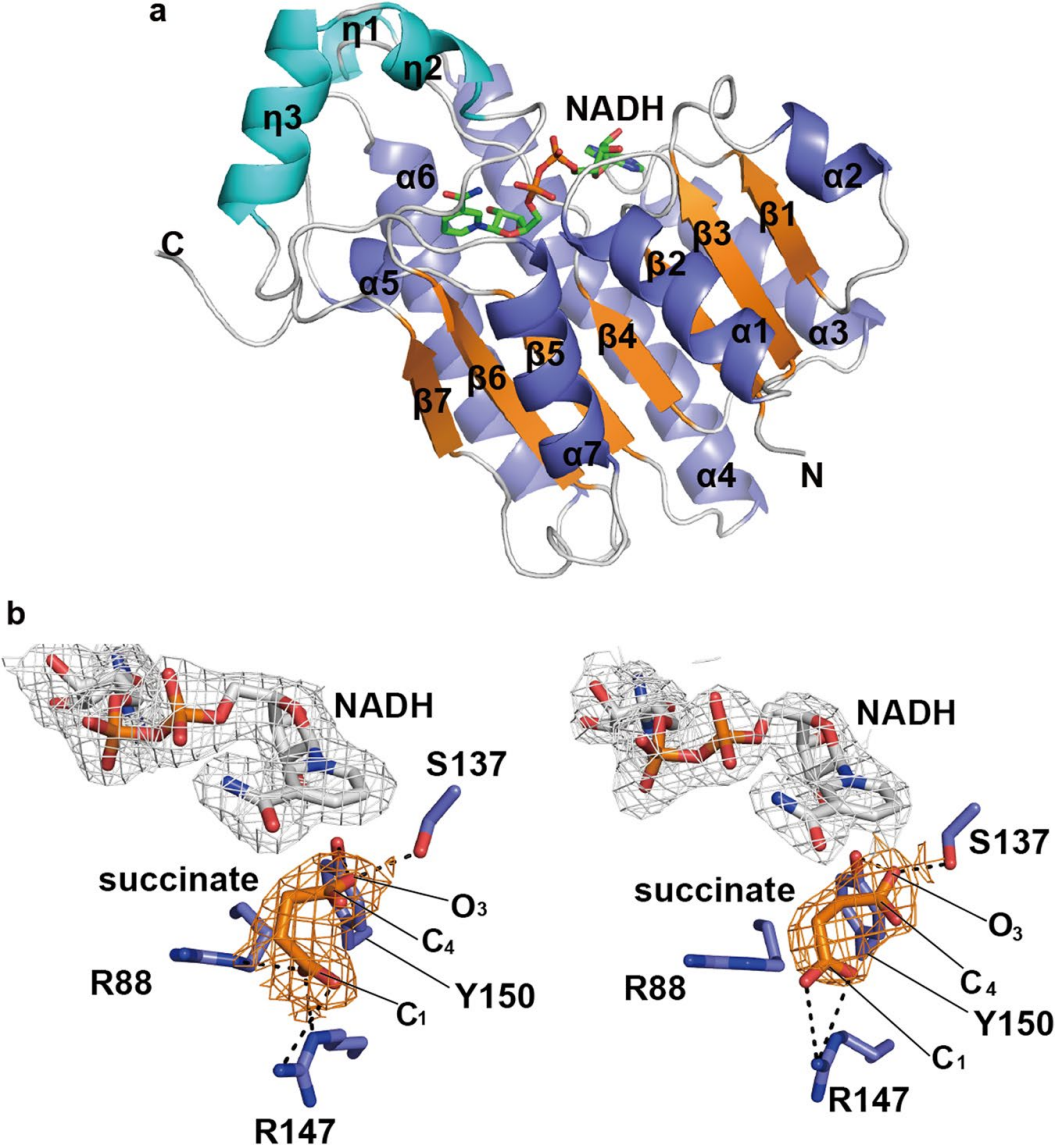
Crystal structure of HILDH in complex with NADH and succinate. (**a**) The core β-strands, α-helices and 3_10_-helices are shown in orange, slate and cyan, respectively. The N- and C-termini are labeled as N and C, respectively. NADH is represented as green sticks. (**b**) *F*_o_ - *F*_c_ electron density omit maps (σ = 1.0) of NADH and succinate bound in chains A (left) and B (right). The chains A and B mean two different subunits of HILDH. In the HILDH-NADH-succinate crystal structure, NADH, succinate and HILDH are shown in white, orange and slate sticks, respectively. Dotted lines show hydrogen bonds or salt bridges.

**Figure 3:**
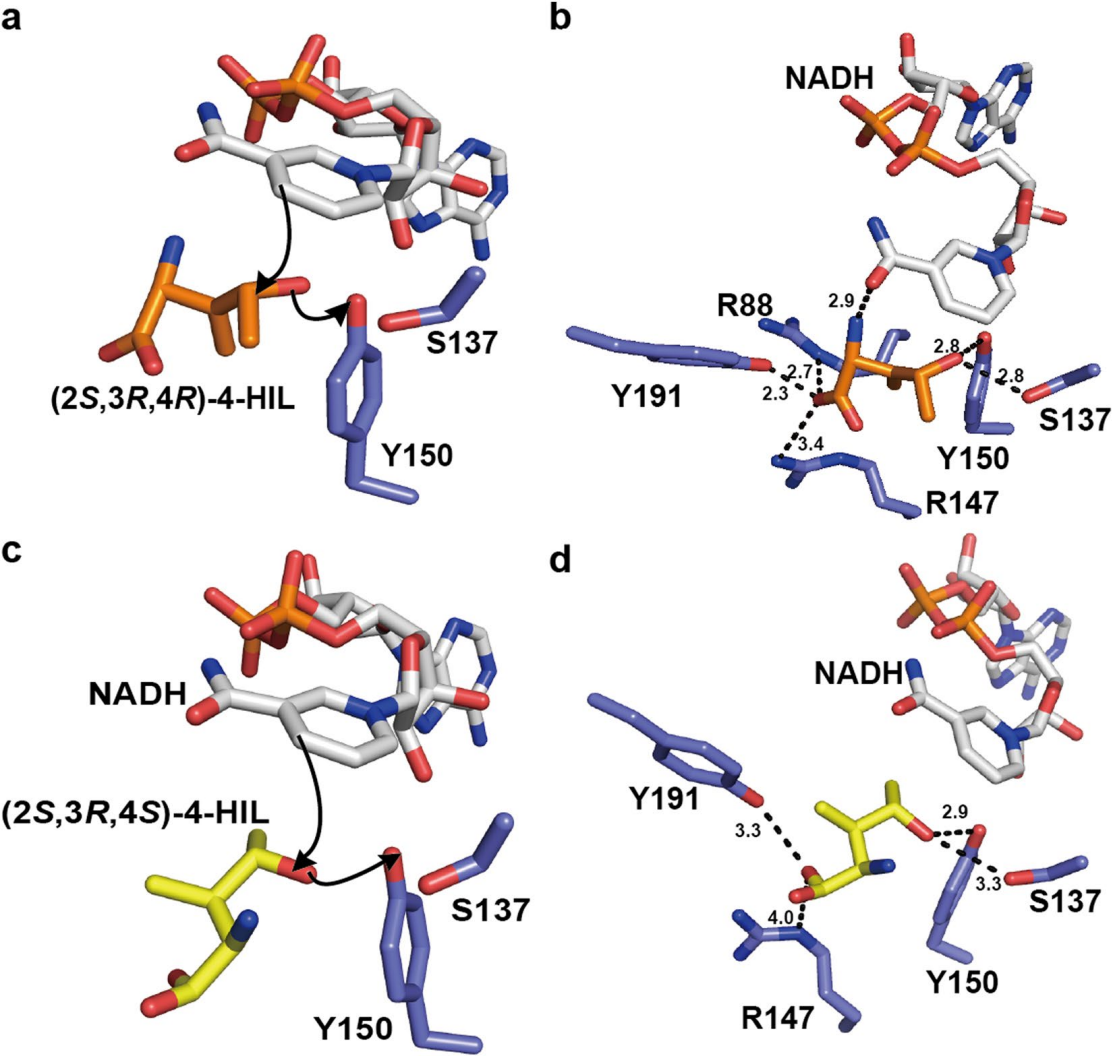
4-HIL-binding mode in the active site of HILDH. (**a**) The putative binding mode of (2*S*,3*R*,4*R*)-4-HIL to HILDH. (2*S*,3*R*,4*R*)-4-HIL, HILDH and NADH are shown in orange, slate and white sticks, respectively. The hydride transfer direction is shown with an arrow. (**b**) The interaction between (2*S*,3*R*,4*R*)-4-HIL and HILDH, where (2*S*,3*R*,4*R*)-4-HIL, HILDH and NADH are shown in orange, slate and white sticks, respectively. Dotted lines show potential hydrogen bonds or salt bridges. Distances are denoted in angstroms. (**c**) The binding model of (2*S*,3*R*,4*S*)-4-HIL constructed by docking simulation; (2*S*,3*R*,4*S*)-4-HIL, HILDH and NADH are shown in yellow, slate and white sticks, respectively. The hydride transfer direction is shown with an arrow. (**d**) The interaction between (2*S*,3*R*,4*S*)-4-HIL and HILDH, where (2*S*,3*R*,4*S*)-4-HIL, HILDH and NADH are shown in yellow, slate and white sticks, respectively. Dotted lines show potential hydrogen bonds or salt bridges. Distances are denoted in angstroms.

The crystal structure of the HILDH-NADH complex has been determined at 2.20 Å resolution. HILDH forms a tetrameric structure, and each subunit consists of seven α-helices, seven β-strands and three 3_10_-helices (Fig. 2a and Supplementary Fig. S2). The subunit structure is divided into two subdomains: the main body and the substrate binding unit. The main body of α/β-folding patterns adopts the Rossmann fold, which is conserved in SDRs. The central β-sheet consists of seven parallel β-strands (β1‒β7) sandwiched between two sets of three parallel α-helices (α1, α2 and α7 on one side and α3, α4 and α6 on the opposite side). The α5 helix and three 3_10_-helices are located on the C-terminal side of the Rossmann fold structure to form the substrate binding unit. The crystal structure of the HILDH-NADH-succinate complex was determined at 2.35 Å resolution. No significant structural differences were found between the structures of HILDH-NADH and HILDH-NADH-succinate, with RMSDs of 0.2-0.3 Å for α-carbons. Succinate, which has structural similarity to 4-HIL, was chosen as a substrate analogue. In the HILDH-NADH-succinate complex, the electron density maps of succinate are observed in chains A and B. The succinate molecule in chain A is bound to the active site of HILDH through 4 hydrogen bonds and a salt bridge (Fig. 2b and Supplementary Table S2). The carboxy group at the C1 position is recognized by R88 and R147, and the carboxy group at C4 position is recognized by S137 and Y150. Additionally, the electron density map of the nicotinamide ring of NADH is observed clearly in each subunit. The reaction mechanism of SDRs requires two types of transfer: a hydride transfer from the nicotinamide ring to the carbonyl group of the substrate and a proton transfer from the catalytic residue Y (Y150 in HILDH) to the carbonyl group of the substrate. In the structure of HILDH-NADH-succinate, the O3 atom of succinate forms hydrogen bonds with S137 and Y150 at 2.8 Å and 2.9 Å, respectively. The C4 atom of nicotinamide is 3.4 Å from the O3 atom of succinate. When the substrate binds to the active site of HILDH with the same binding mode as succinate, a hydride is effectively transferred from the C4 of nicotinamide to the carbonyl group of AMKP. A proton transfer can also take place from catalytic residue Y150 to the carbonyl group of AMKP, making the reduced hydroxyl group face toward Y150. In this reaction mechanism, the reduced hydroxyl group adopts the *R*-form; and the binding mode of succinate mimics that of AMKP to produce the 4*R* form of 4-HIL (Supplementary Fig. S3 and Fig. 3a).

**Figure 4:**
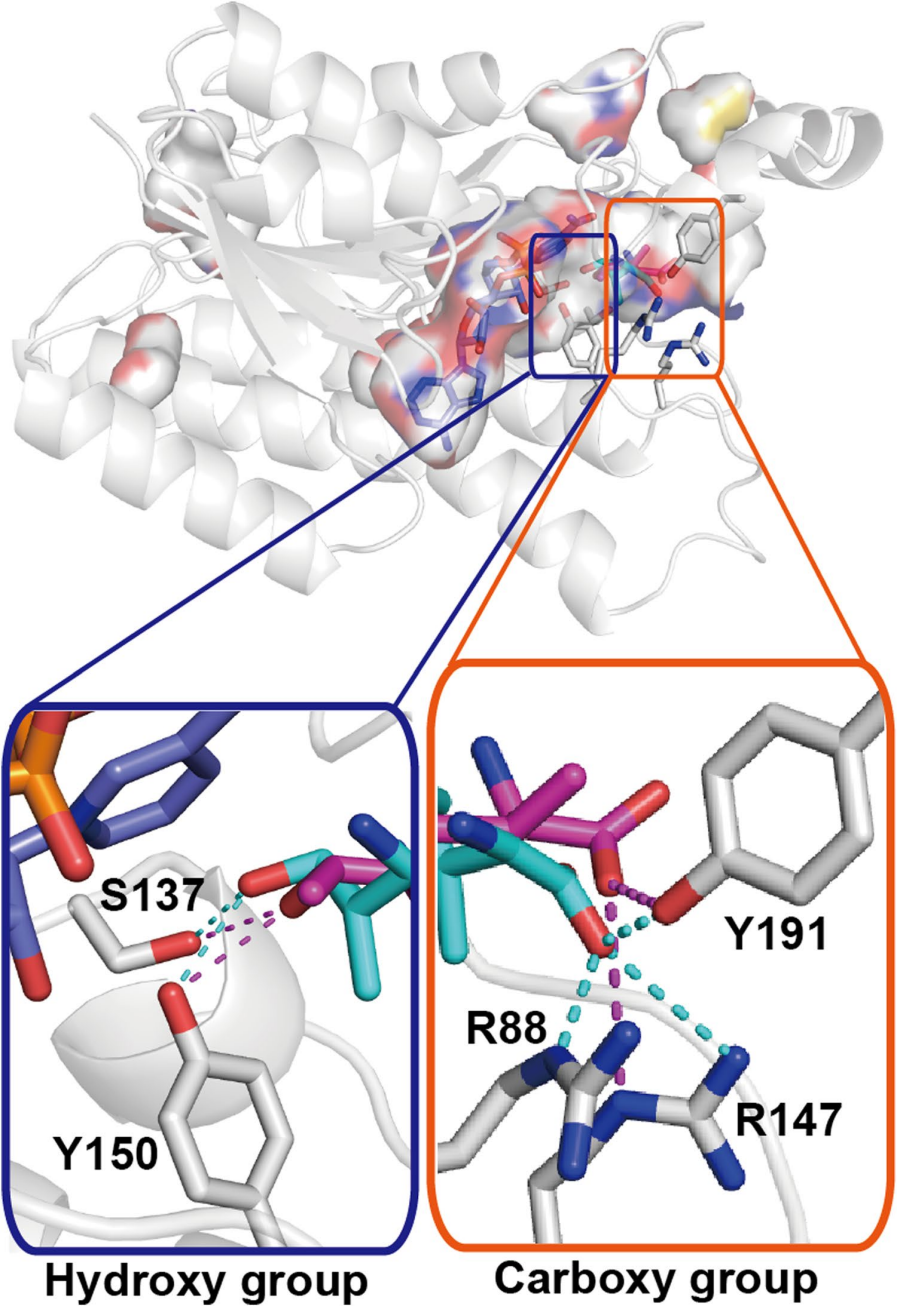
The comparison of (2*S*,3*R*,4*R*)- and (2*S*,3*R*,4*S*)-4-HIL-binding modes. (2*S*,3*R*,4*R*)-4-HIL and (2*S*,3*R*,4*S*)-4-HIL are shown in cyan and magenta sticks, respectively. NADH and HILDH are shown in slate and white sticks, respectively. Dotted lines show potential hydrogen bonds or salt bridges.

To gain insight into the stereoselective reduction of HILDH, we chose (2*S*,3*R*,4*R*)-4-HIL and (2*S*,3*R*,4*S*)-4-HIL as typical 4*R* and 4*S* forms of 4-HIL, respectively, and built two binding models. The binding model of (2*S*,3*R*,4*R*)-4-HIL was built by superimposing the C1 and C4 atoms of (2*S*,3*R*,4*R*)-4-HIL onto the positions of C1 and C4 atoms of succinate in the HILDH-NADH-succinate structure (Supplementary Fig. S3). Figure 3b shows the putative binding mode of (2*S*,3*R*,4*R*)-4-HIL in the active site of HILDH, showing that R88, R147 and Y191 are located within a distance that permits the formation of hydrogen bonds and salt bridge with the carboxy group of (2*S*,3*R*,4*R*)-4-HIL. Because the enzyme reaction is performed at pH 9.1, the side chain of arginine residue is presumed to be positively charged and the carboxy group of substrate/product is presumed to be negatively charged (Supplementary Fig. S4). Thus, R88 is supposed to form salt bridge to recognize the carboxy group of 4-HIL. As shown in Fig. 3b, the 2*S*-amino-group of 4*R*-4-HIL is recognized by the amide oxygen of the nicotinamide ring. Thus, it is supposed that the stereoselectivity toward 2 *S* form is higher than 2*R* form in the 4*R*-4-HIL binding mode. Besides, the 3-methyl group could be form van der Waals contact with the nicotinamide ring of NADH when it adopts 3*S* configuration, supposing that the stereoselectivity toward (2*R*,3*S*,4*R*)-4-HIL is higher than that of (2*R*,3*R*,4*R*)-4-HIL. These assumptions are also supported by high-performance liquid chromatography (HPLC) analysis. As shown in Fig. 1 and Supplementary Table S3, the production ratios of (2*S*,3*R*,4*R*)- and (2*S*,3*S*,4*R*)-4-HIL (14.2% and 13.0%, respectively) are higher than that of (2*R*,3*S*,4*R*)-4-HIL (11.3%) and (2*R*,3*R*,4*R*)-4-HIL (1.3%). The binding model of (2*S*,3*R*,4*S*)-4-HIL was constructed by docking simulation. The lowest-energy docked model in which hydride/proton transfer could take place effectively was chosen as the binding model of (2*S*,3*R*,4*S*)-4-HIL to HILDH (Fig. 3c). As shown in Fig. 3d, the constructed model indicated that the carboxy group of (2*S*,3*R*,4*S*)-4-HIL could interact with R147 and Y191. No residue can directly interact with both binding models of 4-HIL, except for R88, R147 and Y191 and the two catalytic residues S137 and Y150. The distance between R88 and (2*S*,3*R*,4*S*)-4-HIL is more than 4 Å, meaning no ionic or hydrogen bond could be formed. Figure 4 shows the binding mode of (2*S*,3*R*,4*R*)- and (2*S*,3*R*,4*S*)-4-HIL. The carboxy group of 4*S*-4-HIL is recognized by R147 and Y191, while the carboxy group of 4*R*-4-HIL is recognized by R88, R147 and Y191. That is to say, the carboxy groups of 4*S*- and 4*R*-4-HIL are recognized by different residues, forming different carboxy group binding modes. Because R88 is the residue that participates only in the 4*R*-4-HIL recognition, R88 is predicted to be a key residue that enhances the stereoselectivity toward the 4*R*-4-HIL.

Because the substrate used for the reaction is a racemic compound, several types of substrate-binding modes could be formed. The substrate with the carboxy group recognized by R88, R147 and Y191 would be reduced by 4*R*-4-HIL. The substrate with the carboxy group recognized by R147 and Y191 would be reduced by 4*S*-4-HIL. These recognition modes were presumed based on the product-binding modes that show different recognition patterns of the carboxy group of products. Thus, the product binding modes are more suitable for discussion.

### Residues recognizing the C4-Carboxy group of AMKP control the stereoselective reduction of HILDH

**Figure 5:**
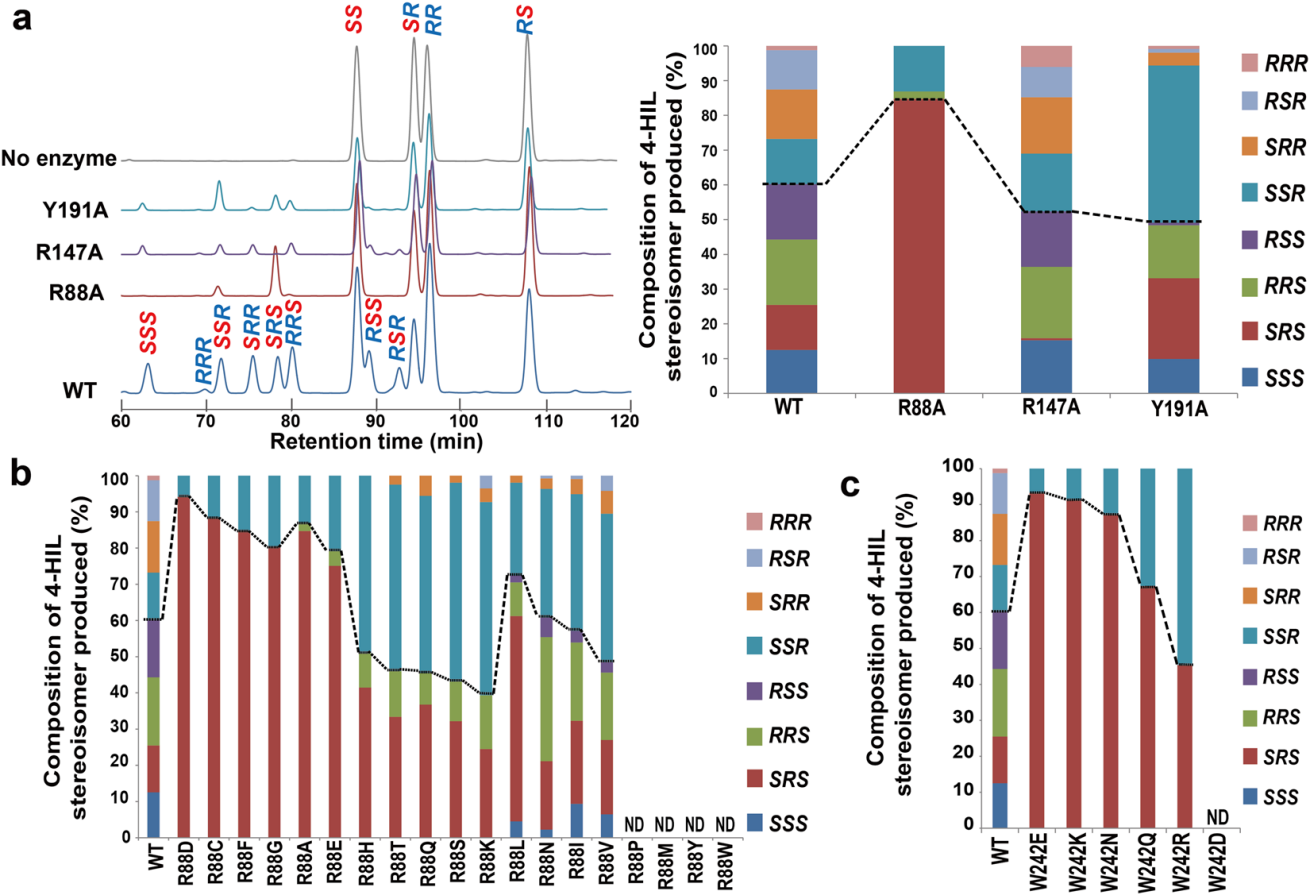
HPLC analysis of 4-HIL stereoisomers produced by the wild-type (WT) and the mutant HILDH. (**a**) HPLC chromatogram (left) and bar graph (right) of 4-HIL stereoisomers produced by the wild-type HILDH and the mutants of R88, R147 and Y191. “No enzyme” represents the reaction mixtures without HILDH. The reaction mixtures were derivatized with GITC and detected by HPLC. For all rows, dotted line in bar graph divides products into 4*S* forms (below the dotted line) and 4*R* forms (above the dotted line) of 4-HIL. (**b**) 4-HIL stereoisomers produced by the R88 mutants. ND means not detected by HPLC analysis. Dotted line divides products into 4*S* forms (below the dotted line) and 4*R* forms (above the dotted line) of 4-HIL. (**c**) 4-HIL stereoisomers produced by the W242 mutants. ND means not detected by HPLC analysis. Dotted line divides products into 4*S* forms (below the dotted line) and 4*R* forms (above the dotted line) of 4-HIL.

Based on the binding models of 4-HIL, we prepared the A mutants of R88, R147 and Y191 and measured their catalytic activities. The kinetic parameters are summarized in Table 1. Compared to the wild type, R88A and Y191A mutants showed very low activities (4% and 6%, respectively). The R147A mutant retained 32% activity and the catalytic efficiency (*k*_cat_/*K*_m_) was 40.7% of the wild type, mainly due to the lower *k*_cat_. These results suggest that the R88A, R147A and Y191A mutants affect enzyme activity in the reduction of AMKP. These results also suggest that the residues recognizing the C4-carboxy group of 4-HIL contributed to substrate recognition. To assess the effect of these residues on stereoselectivity, the 4-HIL stereoisomers produced by the wild type and mutants were measured by HPLC. The yield and composition of each 4-HIL stereoisomer are summarized in Supplementary Table S3 and Fig. 5a. The HPLC analysis showed that HILDH reduced the racemic AMKP to 4-HIL with loose stereoselectivity, producing eight 4-HIL stereoisomers, including (2*S*,3*R*,4*S*)-4-HIL in the production ratio of 13.0%. In the wild type, the production ratios of the seven 4-HIL stereoisomers except (2*R*,3*R*,4*R*)-4-HIL were nearly the same (11.3–18.8%). The production ratios of the 4*R* and 4*S* forms of 4-HIL were 39.7% and 60.3%, respectively. In the R88A mutant, the production ratio of the 4*R* form decreased to 13.1% and the production ratio of 4*S* form increased to 86.9%. This indicates that R88 plays an important role in recognizing the 4*R* form of 4-HIL, consistent with structural inspection of the 4-HIL-binding models. The production ratios of the 4*R* and 4*S* forms of 4-HIL in the R147A and Y191A mutants were similar to that of wild type (1:1.1 and 1:0.97, respectively). However, the production ratio of each 4-HIL stereoisomer changed. In the R147A mutant, the production of the 2*S*,3*R*,4*S* form fell to 0.6%, indicating that R147 is required to maintain the productivity of (2*S*,3*R*,4*S*)-4-HIL. On the other hand, the Y191A mutation increased the production ratio of the 2*S*,3*S*,4*R* form to 45.2% and decreased the 2*R*,3*S*,4*S* and 2*R*,3*S*,4*R* forms to 0.8% and 1.0%, respectively, indicating that substitution of Y191 for A led to a decrease in stereoselectivity toward 2*R*,3*S*-AMKP.

**Table 1:** NADH-dependent activity and kinetic parameters of the wild-type and the mutant HILDH.

Enzyme	Specific activity (U/mg)^a^	*K*_m_ (mM)^a^	*k*_cat_^a^(s^−1^)^a^	*k*_cat_/ *K*_m_ (s^−1^ mM^−1^)
Wild type	564.5 ± 12.9	2.0 ± 0.5	532.3 ± 65.1	266.2
R88A	25.0 ± 14.5	ND^b^	ND^b^	ND^b^
R147A	181.1 ± 13.1	2.3 ± 0.9	249.2 ± 47.5	108.4
Y191A	31.6 ± 4.5	ND^b^	ND^b^	ND^b^

^a^Mean ± standard error (*n* = 3).

^b^ND means not detected.

Although we failed to obtain a substrate- or product-binding structure, the results of site-directed mutagenesis support the validity of the 4-HIL-binding model. Thus, we started protein engineering of HILDH to create a novel engineered AMKP reductase that stereoselectively produces (2*S*,3*R*,4*S*)-4-HIL. Because the R88A mutation showed the most significant improvement in selectivity toward (2*S*,3*R*,4*S*)-4-HIL, we performed site-saturation mutagenesis of R88 for the first round of enzyme engineering. The result of HPLC analysis is shown in Fig. 5b and Supplementary Table S4. The R88D mutant produced only two 4-HIL diastereomers, (2*S*,3*R*,4*S*)- and (2*S*,3*S*,4*R*)-4-HIL. The purity of (2*S*,3*R*,4*S*)-4-HIL was 94.2%, making this mutant the most stereoselective. Similar selectivity changes were also observed in the R88C, R88F and R88G mutants, producing (2*S*,3*R*,4*S*)-4-HIL at purities of 88.3%, 84.7% and 80.4%, respectively. The increased stereoselectivity for 4*S* was also observed in the R88A, R88E R88L and R88N mutants. The R88H, R88T, R88Q, R88S, R88K, R88I and R88V mutants showed lower stereoselectivity for 4*S* as compared with the wild type. These results suggest that acidic and some hydrophobic residues decrease the production of 4*R* by weakening its interaction with the C4-carboxy group of 4-HIL.

### Improved stereoselectivity toward (2*S*,3*R*,4*S*)-4-HIL by the combination of mutagenesis

**Figure 6:**
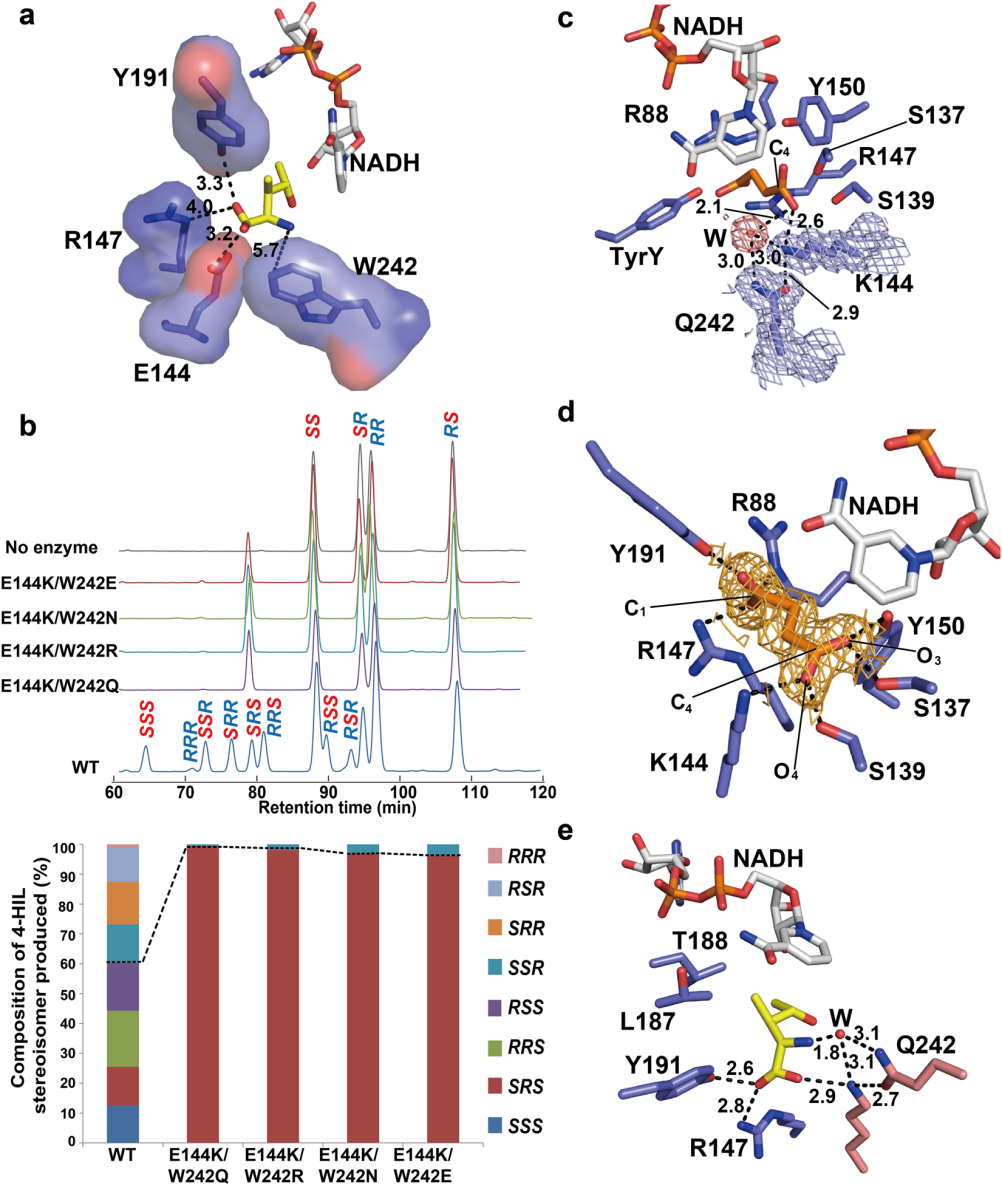
Improved stereoselectivity of the E144/W242 double mutant toward (2*S*,3*R*,4*S*)-4-HIL. (**a**) Binding model of (2*S*,3*R*,4*S*)-4-HIL to the active site of HILDH. (2*S*,3*R*,4*S*)-4-HIL, HILDH and NADH are shown in yellow, slate and white sticks. Distances in angstroms are denoted by dotted lines. (**b**) HPLC chromatogram (upper) and bar graph (lower) of 4-HIL stereoisomers produced by the wild type (WT) and the double mutants of E144 and W242. “No enzyme” indicates the reaction mixtures without HILDH. The reaction mixtures were derivatized with GITC and detected by HPLC. Dotted line in bar graph divides products into 4*S* forms (below the dotted line) and 4*R* forms (above the dotted line) of 4-HIL. (**c**) *F*_o_ - *F*_c_ electron density omit map (σ = 1.0) of K144 and Q242 of HILDH^E144K/W242Q^-NADH- succinate complex. Succinate, HILDH and NADH are shown in orange, slate and white sticks, respectively. Red spheres represent water molecules. Dotted lines show potential hydrogen bonds or salt bridges. Distances are denoted in angstroms. (**d**) *F*_o_ - *F*_c_ electron density omit map (σ = 1.0) of succinate bounded in the structure of HILDH^E144K/W242Q^-NADH- succinate complex. Succinate, HILDH and NADH are shown in orange, slate and white sticks, respectively. Dotted lines show hydrogen bonds or salt bridges. (**e**) The binding model of (2*S*,3*R*,4*S*)-4-HIL to HILDH^E144K/W242Q^ constructed by docking simulation. K144 and Q242 are shown in pink sticks. (2*S*,3*R*,4*S*)-4-HIL, HILDH and NADH are shown in yellow, slate and white sticks, respectively. Red spheres represent water molecules. Dotted lines show potential hydrogen bonds or salt bridges. Distances are denoted in angstroms.

To further improve stereoselectivity toward (2*S*,3*R*,4*S*)-4-HIL, 2*S*-amino-group recognition by polar amino acids may be required in addition to interaction with the C4-carboxy group. Among the amino acid residues located near (2*S*,3*R*,4*S*)-4-HIL, we chose W242 and E144 as candidate residues for protein engineering (Fig. 6a). W242 is located near the 2*S*-amino group of (2*S*,3*R*,4*S*)-4-HIL, with a distance of 5.7 Å. E144 is located near the carboxy group of (2*S*,3*R*,4*S*)-4-HIL with a distance of 3.2 Å, and may interfere with 4-HIL binding to produce the 4*S* form. We have confirmed that the amino group of 4-HIL is protonated at pH 9.1 (Supplementary Fig. S4). Because the distance between W242 and the amino group of 4-HIL is 5.7 Å, we presumed that W242 mutation to polar amino acids with long side chains may form water-mediated hydrogen bonds with the amino group or/and carboxy group of (2*S*, 3*R*, 4*S*)-4-HIL. We evaluated stereoselectivity changes induced by the W242E/K/N/Q/R/D mutations. All mutations except for the W242D mutation enhanced selectivity toward (2*S*,3*R*,4*S*)-4-HIL (Fig. 5c and Supplementary Table S5). Among them, the W242E mutant showed the highest stereoselectivity toward (2*S*,3*R*,4*S*)-4-HIL and produced (2*S*,3*R*,4*S*)-4-HIL at a purity of 86.5%. In the first round of enzyme engineering, the R88D, R88C, R88F and R88G mutants showed high stereoselectivity toward (2*S*,3*R*,4*S*)-4-HIL. Using these four R88 mutants as template enzymes, we constructed double mutants by substituting W242 with E, K, N, Q and R. When the reaction product of each mutant was analyzed by HPLC, the R88C/W242N, R88C/W242E, R88C/W242K, R88C/W242Q and R88G/W242R mutants produced two 4-HIL diastereomers: (2*S*,3*R*,4*S*)- and (2*S*,3*S*,4*R*)-4-HIL (Supplementary Table S6). The R88G/W242E, R88G/W242N and R88G/W242Q mutants produced three 4-HIL stereoisomers: (2*S*,3*R*,4*S*)-, (2*R*,3*R*,4*S*)- and (2*S*,3*S*,4*R*)-4-HIL, and other mutants showed no activity (data not shown). The R88C/W242N mutant showed the highest stereoselectivity toward (2*S*,3*R*,4*S*)-4-HIL, producing (2*S*,3*R*,4*S*)-4-HIL at a purity of 90.8%. Because the stereoselectivity of the double mutant is lower than those of each single mutant at R88 and W242, other mutational combinations are required to improve the stereoselectivity toward (2*S*,3*R*,4*S*)-4-HIL.

To enhance the interaction with the C4-carboxy group in the (2*S*,3*R*,4*S*)-4-HIL-binding model, we constructed double and triple mutants of HILDH by substituting E144 to R or K in the R88 single mutants (R88D, R88C, R88F and R88G), the W242 single mutants (W242E, W242K, W242N, W242Q and W242R) and the R88/W242 double mutants (R88C/W242N, R88C/W242E, R88C/W242K, R88C/W242Q, R88G/W242R). All triple mutants showed no activity. On the other hand, E144K/W242E, E144K/W242N, E144K/W242Q and E144K/W242R produced two types of 4-HIL diastereomers: (2*S*,3*R*,4*S*)-4-HIL as a main product and a small amount of (2*S*,3*S*,4*R*)-4-HIL as a minor product (Fig. 6b and Supplementary Table S7). Among them, the E144K/W242Q double mutant produced (2*S*,3*R*,4*S*)-4-HIL at a purity of 99.1%, high enough optical purity for industrial use. The yield of (2*S*,3*R*,4*S*)-4-HIL after 24 h reaction in the E144K/W242Q mutant (151.9 ± 43.3 μg/ml) was comparable to that of wild-type (95.2 ± 10.3 μg/ml), suggesting that these mutations did not affect the production yield of (2*S*,3*R*,4*S*)-4-HIL.

### Structural basis of strict stereoselectivity toward (2*S*,3*R*,4*S*)-4-HIL

To explain the structural mechanism of the high selectivity of the E144K/W242Q mutant toward (2*S*,3*R*,4*S*)-4-HIL, we solved the crystal structure of HILDH^E144K/W242Q^ in complex with NADH and succinate at 1.90 Å resolution. The electron densities of succinate molecules, K144 and Q242 are clearly observed in the active site of all four subunits to HILDH^E144K/W242Q^ (Fig. 6c and d). The electron density of the K residue is often obscured because the side-chain is flexible. However, in the crystal structure of HILDH^E144K/W242Q^, the electron density of K144 is clearly observed; the side-chain configuration of K144 is fixed by a hydrogen-bonding network with succinate, Q242 and a water molecule. The side-chain amino group of K144 forms a hydrogen bond with the side-chain amide oxygen of Q242. A water molecule is trapped by the side-chain amino group of K144, the side-chain amide nitrogen of Q242 and the C1-carboxy group of succinate. The succinate molecule bound to the active site of HILDH^E144K/W242Q^ is recognized by 7 residues of HILDH^E144K/W242Q^ (Fig. 6d). The C4-carboxy group interacts with S137, S139, K144 and Y150. The carboxy group at C-1 is recognized by R88, R147 and Y191. The binding mode of succinate in the HILDH^E144K/W242Q^ mutant mimics that of the 4*R* form of 4-HIL, as well as the wild-type structure. The O3 atom of succinate forms hydrogen bonds with two catalytic residues: S137 and Y150. The O4 atom is recognized by S139 and K144, but not when the O4 atom is substituted by a methyl group in the 4*R* form of 4-HIL, the hydrophobic methyl group would hardly be adopted by hydrophilic S139 and K144. Therefore, the mutated K144 and Q242 play critical roles in decreasing stereoselectivity toward the 4*R* form of 4-HIL.

The binding model of (2*S*,3*R*,4*S*)-4-HIL to HILDH^E144K/W242Q^ was built by docking simulation in the same way as the wild-type HILDH (Fig. 6e). The side-chain amino group of K144 can form a salt bridge with the C4-carboxy group of (2*S*,3*R*,4*S*)-4-HIL. Together with the side chain of Q242, K144 forms a hydrogen-bonding network through a water molecule that is used to recognize the 2*S*-amino group and C4-carboxy group of (2*S*,3*R*,4*S*)-4-HIL. Furthermore, the binding model showed that the 3*R*-methyl group of (2*S*,3*R*,4*S*)-4-HIL favorably faced the hydrophobic pocket formed by the nicotinamide ring of NADH, L187 and T188. These interactions allow (2*S*,3*R*)-AMKP to specifically bind the active site, with the orientation enabling the substrate to be reduced to the 4*S* form. The HILDH^E144K/W242Q^ mutant produced (2*S*,3*R*,4*S*)-4-HIL with strict stereoselectivity (>99% de), and the production yield of (2*S*,3*R*,4*S*)-4-HIL after 24 h reaction was the same as the wild type, despite it showed 4% enzyme activity compared with the wild type. Compared to engineered enzymes, naturally evolved enzymes may be highly optimized through selection over millions of years. We used the Basic Local Alignment Search Tool (BLAST) to search for proteins carrying mutations similar to HILDH^E144K/W242Q^. We found no native enzyme possessing residues that determined stereoselectivity of HILDH^E144K/W242Q^ toward (2*S*,3*R*,4*S*)-4-HIL, namely K144, R147, L187, T188, Y191 and Q242 of HILDH^E144K/W242Q^ were not found by BLAST search (Supplementary Table S8).

## Conclusions

Fenugreek seeds contain nutrients including β-carotene, ascorbate, fiber, vitamins and amino acids and are thus one of the most ancient medicinal herbs and an important dietary supplement for anti-aging and human health. To overcome the biggest limitation to commercial scale production of (2*S*,3*R*,4*S*)-4-HIL, we applied protein engineering approach to modify the key enzyme 4-HIL dehydrogenase (HILDH). HILDH reduces the racemic AMKP to 4-HIL with loose stereoselectivity, producing seven 4-HIL stereoisomers, including (2*S*,3*R*,4*S*)-4-HIL in a production ratio of 13%. Based on the structural insights obtained from the crystal structure and 4-HIL-binding models of HILDH, we succeeded in generating the E144K/W242Q double mutant (HILDH^E144K/W242Q^) via a protein engineering approach. The crystal structure of HILDH^E144K/W242Q^ was solved to elucidate the structural mechanism of its strict stereoselectivity toward (2*S*,3*R*,4*S*)-4-HIL. The binding model of (2*S*,3*R*,4*S*)-4-HIL to HILDH^E144K/W242Q^ suggests that mutated K144 and Q242 could not only strengthen the stereoselective recognition of (2*S*,3*R*,4*S*)-4-HIL but also decrease stereoselectivity toward the 4*R* form of 4-HIL. The engineered enzyme proposed in this study is expected to be used in industrial synthesis of (2*S*,3*R*,4*S*)-4-HIL. Furthermore, the structure-based protein engineering approach of HILDH could be useful for the synthesis of other 4-HIL stereoisomers, including (2*S*,3*S*,4*R*)-4-HIL, which is extracted from *Quararibea funebris* flowers and used as a spice in Mexico^54^. This stereoisomer is also used in local medicine to control cough, fevers, menstrual disorder and psychopathic fears. Our innovative and effective method of synthesizing (2*S*,3*R*,4*S*)-4-HIL by modifying HILDH is expected to lead to its mass production and contribute to drug development for its application to various diseases including diabetes. Further research would be needed in separating starting materials or using other racemic compound for commercial use. However, we expect that the structure-based protein engineering approach discussed in this study would be of general interest in protein engineering especially for asymmetric reductase.

## Materials and Methods

### Protein preparation and crystallization

The HILDH-coding gene from *B. thuringiensis* 2e2 was amplified by polymerase chain reaction (PCR) with two primers, 5′-CACCATGAGAGAGAATAAAATAATTATGA-3′ and 5′-CTCGAGCTACAAGTTTTTCCCAGCAGTCCAA-3′, and cloned into the pET101/D-TOPO vector (Invitrogen). The recombinant protein had an additional sequence at the C terminus (LEKGELNSKLEGKPIPNPLLGLDSTRTGHHHHHH) that contains a V5 epitope and a hexahistidine tag (both shown by underline). *Escherichia coli* Rosetta (DE3) cells harboring the expression plasmid pET101-HILDH were grown at 37 °C in lysogeny broth (LB) medium until the OD_600_ reached 0.6‒0.8. Protein expression was induced by addition of isopropyl β-D-1-thiogalactopyranoside (IPTG) at a final concentration of 0.5 mM, and the culture was further incubated for 18 h at 25 °C. After harvesting, the cells were lysed by sonication in lysis buffer containing 20 mM Tris-HCl (pH 7.4), 0.5 M NaCl, 30 mM imidazole and 1 mM dithiothreitol (DTT), and the disrupted cells were centrifuged at 40,000 × *g* for 30 min. The supernatant was purified using Ni-NTA Superflow resin (Qiagen, Tokyo, Japan) and an elution buffer containing 20 mM Tris-HCl (pH 7.4), 0.5 M NaCl, 200 mM imidazole and 1 mM DTT. After dialysis against 20 mM Tris-HCl (pH 8.5) and 1 mM DTT, the sample was applied to a Resource Q (GE Healthcare Tokyo, Japan) column and eluted with a linear gradient of 100−400 mM NaCl. The fractions containing HILDH were further purified using a Superdex 200 (GE Healthcare Tokyo, Japan) column equilibrated with 20 mM Tris-HCl (pH 7.4), 300 mM NaCl and 1 mM DTT. Pooled fractions were dialyzed against 20 mM Tris-HCl (pH 7.4) and were concentrated to 15 mg/ml for crystallization.

HILDH was crystallized using the sitting-drop vapor diffusion method at 20 °C. NADH-bound crystals were obtained by mixing 1.0 μl protein (10 mg/ml) containing 10 mM NADH with 1.0 μl reservoir solution consisting of 30% (*w/v*) PEG 400, 0.1 M acetate (pH 4.5) and 0.2 M calcium acetate. Crystals of HILDH complexed with NADH and succinate were obtained by mixing 1.0 μl protein (10 mg/ml) containing 10 mM NADH and 100 mM succinic acid with 1.0 μl reservoir solution consisting of 2.0 M ammonium sulfate, 0.1 M citrate (pH 5.5).

The expression plasmids for the HILDH E144K/W242Q mutant (HILDH^E144K/W242Q^) were generated by site-directed mutagenesis. PCR was carried out using PrimeSTAR Max DNA polymerase (Takara Bio., Shiga, Japan) and the expression plasmid pET101-HILDH as a template. Mutations were confirmed by DNA sequencing (Fasmac Co., Ltd, Kanagawa, Japan). Mutant enzyme was purified according to the same method as the wild-type enzyme. Crystals of HILDH^E144K/W242Q^ bound with NADH and succinate were obtained by mixing 1.0 μl protein (8 mg/ml) containing 10 mM NADH with 1.0 μl reservoir solution (0.8 M succinic acid, pH 7.0).

### Data collection, processing and structure analysis

X-ray diffraction data sets of the HILDH-NADH complex and HILDH-NADH-succinate complex were collected at the BL-5A beamline at the Photon Factory (Tsukuba, Japan). X-ray diffraction data of the HILDHE144K/W242Q-NADH-succinate complex was collected on the BL-17A beamline at the Photon Factory. All diffraction data sets were indexed, integrated and scaled with HKL-2000^55^ and XDS^56^. The structure of HILDH-NADH was determined using the molecular replacement method performed by the program MOLREP^57^ on the CCP4 suite using the structure of 3-ketoacyl-(acyl-carrier-protein) reductase (PDB code, 3F9I; sequence identity, 34%)^58^ as the initial model. The structures of HILDH-NADH-succinate and HILDHE144K/W242Q-NADH- succinate were determined by the molecular replacement method using the structure of HILDH-NADH as the initial model. Refinement was performed with COOT^59^ and Refmac^60^. The data collection and processing statistics are summarized in Supplementary Table S1. The structure coordinates and structural factors have been deposited in the Protein Data Bank under accession codes 5GWR (HILDH-NADH form), 5GWS (HILDH-NADH-succinate form) and 5GWT (HILDHE144K/W242Q-NADH- succinate form).

### Model building of 4-HIL to the active site of HILDH

Because the binding mode of succinate to the active site of HILDH mimics the 4*R* form of 4-HIL in the crystal structure of the HILDH-NADH-succinate complex, we chose (2*S*,3*R*,4*R*)-4-HIL as a typical 4*R* form of 4-HIL and constructed the binding model of (2*S*,3*R*,4*R*)-4-HIL to HILDH based on succinate binding using PyMol (http://pymol.sourceforge.net). The (2*S*,3*R*,4*R*)-4-HIL molecule was generated using the Molecular builder of the Molecular Operating Environment software package (MOE, Ryoka Systems Inc., Montreal, Canada). The C_1_ and C_4_ atoms of 4-HIL were superimposed onto the C_1_ and C_4_ atoms of succinate, respectively, and the hydroxyl group of (2*S*,3*S*,4*R*)-4-HIL was positioned to face toward the hydroxyl group of Y150, so that effective hydride transfer can occur.

The (2*S*,3*R*,4*S*)-4-HIL binding model was built by docking simulation using MOE software. All water molecules in the HILDH-NADH complex were removed and the hydrogen atoms were generated using the Protonate3D program at pH 9.1. The amino group of 4-HIL was protonated and the carboxy group of 4-HIL was deprotonated (Supplementary Fig. S4). Partial charges of all atoms were calculated, and rigid-body energy minimization was used under the MMFF94x (Merck molecular force field 94x) force field. The Site Finder module was used to find the potential binding site of (2*S*,3*R*,4*S*)-4-HIL, which was generated with the Molecular builder of MOE. Docking simulation was performed using the ASEDock module. The docking models were searched with the following parameters: methodology, LowModeMD which is appropriate to generate conformations of small molecules and protein loops; cutoff, 4.5 Å; RMS gradient, 10 kcal/mol/Å and energy threshold, 500 kcal/mol/Å. Docking results of complex structures were sorted by the rank of U_dock score [U_ele (electric energy) + U_vdw (van der Waals energy) + U_solv (Solvation energy) + U_strain (Strain energy)] (kcal/mol)] (Supplementary Fig. S5). We selected the binding model by two criteria: i) Model shows low U_dock score (means energetically stable) and ii) hydride/proton transfer could take place in the model. Among the docking models of the wild type HILDH, the model 1 shows the lowest U_dock score and the hydride/proton transfer could take place (Supplementary Fig. S6). Therefore, we selected the model 1 (PDB file 2) as the binding model of (2*S*,3*R*,4*S*)-4-HIL to the wild type (also shown in Fig. 3c). The binding model of (2*S*,3*R*,4*S*)-4-HIL to the E144K/W242Q mutant HILDH was built in the same way as the wild-type HILDH (Supplementary Fig. S7). Among the docking models, the model 1 shows the lowest U_dock score. However, hydride/proton transfer could not take place in the model 1 (Supplementary Fig. S8). We selected the model 4 (PDB file 3), which shows the fourth lowest U_dock score and where hydride/proton transfer could take place.

### Preparation of HILDH and its mutants for enzyme assay

Expression plasmids for HILDH mutants were generated by site-directed mutagenesis. PCR was carried out using PrimeSTAR Max DNA polymerase (Takara Bio., Shiga, Japan) and the expression plasmid pET101-HILDH as a template. Mutations were confirmed by DNA sequencing (Fasmac Co., Ltd, Kanagawa, Japan). The Wild-type and mutant enzymes were prepared by the following steps. Culture supernatants were lysed by sonication in lysis buffer containing 20 mM Tris-HCl (pH 7.4), 0.5 M NaCl, 30 mM imidazole and 1 mM DTT, and then centrifuged. The supernatant was purified using Ni-NTA Superflow resin (Qiagen, Tokyo, Japan). The protein was eluted with elution buffer containing 20 mM Tris-HCl (pH 7.4), 0.5 M NaCl, 200 mM imidazole and 1 mM DTT, dialyzed against 50 mM Tris-HCl (pH 9.1) and concentrated (~10 mg/ml) for enzyme assay and product analysis.

### Enzyme assay

Enzyme activity was measured by detecting the decrease in absorbance of NADH at 340 nm using a Shimadzu UV-2450 spectrophotometer (Shimadzu, Japan). The reaction solution contained 30 μg/ml enzyme, 2 mM AMKP and 0.2 mM NADH and 50 mM Tris-HCl (pH 9.1). The reaction was performed at 37 °C for 2 min. The initial rate for AMKP were determined using different concentrations (2 mM, 2.5 mM, 3 mM) of AMKP in the presence of 0.2 mM NADH. One unit was defined as the amount of enzyme catalyzing 1.0 nmol of substrate per minute. The kinetic parameters were determined by Lineweaver-Burk plot using the initial rate. All assays were performed at least three times. The *K*_m_ and *k*_cat_ values were calculated using SigmaPlot 13.0 (Systat Software Inc., Chicago, IL.).

### Product analysis

The reaction solution contained 0.2 mM enzyme, 2 mM AMKP, 27 mM NADH and 50 mM Tris-HCl (pH 9.1). The reaction was performed at 37 °C for 24 h. For product analysis, reaction mixtures were derivatized with 2,3,4,6-tetra-*O*-acetyl-β-D-glucopyranosyl isothiocyanate (GITC). Reaction mixtures were mixed with the same volume of trimethylamine (1.8% in acetonitrile) and two volumes of GITC (10 mg/ml in acetonitrile) and were reacted for 30 min at 25 °C. The amino acid derivatives were analyzed by HPLC on a Shimadzu LC-10ATvp HPLC system (Shimadzu, Kyoto, Japan) equipped with two CAPCELLC_18_ Type MG columns (Shiseido, Tokyo, Japan, 4.6 × 250 mm) at a flow rate of 1 ml/min at 40 °C, with 254 nm UV detection. Solvent gradients were as follows: solution A, 10 mM KH_2_PO_4_ (pH 2.95); and solution B, 100% acetonitrile. The elution was performed with a linear gradient from 80% solution A/20% solution B to 73% solution A/27% solution B. Retention time of AMKP and 4-HIL stereoisomers were determined from the injection of each synthesized stereoisomer. HPLC chromatogram of four stereoisomers of AMKP and eight stereoisomers of 4-HIL to indicate the retention time of each stereoisomer was show in Supplementary Figure S9. The mixtures of racemic AMKP and racemic 4-HIL were derivatized with GITC and detected by HPLC. The derivatized (2*R*,3*R*,4*R*)-4-hydroxyisoleucine was separately detected from other 4-HIL stereoisomers and AMKP. The amount of each stereoisomer of 4-HIL was estimated on the basis of comparison with a (2*S*,3*R*,4*S*)-4-HIL standard.

## Electronic supplementary material

Supplementary Information
