# Supplementary material for: Engineering a short-chain dehydrogenase/reductase for the stereoselective production of (2S,3R,4S)-4-hydroxyisoleucine with three asymmetric centers
Source: Sci Rep. 2017 Oct 20;7:13703. doi: 10.1038/s41598-017-13978-w (PMC5651801; doi:10.1038/s41598-017-13978-w)
Supplement: Supplementary file 1 — Supplementary Information [file 41598_2017_13978_MOESM1_ESM.pdf]

Supplementary Information

Engineering a short-chain dehydrogenase/reductase for the stereoselective production of (2*S*,3*R*,4*S*)-4-hydroxyisoleucine with three asymmetric centers

Xuan Shi, Takuya Miyakawa, Akira Nakamura, Feng Hou, Makoto Hibi, Jun Ogawa, Yeondae Kwon, Masaru Tanokura*

*Corresponding author. E-mail: amtanok@mail.ecc.u-tokyo.ac.jp

Contents

Supplementary Text: Structure determination of the HILDH-NADH, HILDH-NADH-succinate and HILDH$^{E144K/W242Q}$-NADH-succinate complexes

Supplementary Table S1: Data collection and refinement statistics

Supplementary Table S2: Hydrogen bonds and salt bridges between HILDH and succinate

Supplementary Table S3: 4-HIL stereoisomers produced by the wild-type and mutant HILDH

Supplementary Table S4: 4-HIL stereoisomers produced by the R88 mutants

Supplementary Table S5: 4-HIL stereoisomers produced by the W242 mutants

Supplementary Table S6: 4-HIL stereoisomers produced by the R88/W242 double mutants

Supplementary Table S7: 4-HIL stereoisomers produced by the E144/W242 double mutants

Supplementary Table S8: Multiple sequence alignment of HILDH$^{E144K/W242Q}$ mutant and SDR homologues

Supplementary Figure S1: Eight stereoisomers of 4-hydroxyisoleucine (4-HIL)

Supplementary Figure S2: Overall structure of HILDH

Supplementary Figure S3: Superimposition of (2*S*,3*R*,4*R*)-4-HIL onto succinate

Supplementary Figure S4: Simulated protomer/tautomer states at pH 9.1

Supplementary Figure S5: Docking results of (2*S*,3*R*,4*S*)-4-HIL (wild-type HILDH)

Supplementary Figure S6: Docking models of (2*S*,3*R*,4*S*)-4-HIL (wild-type HILDH)

Supplementary Figure S7: Docking results of (2*S*,3*R*,4*S*)-4-HIL (HILDHE$^{144K/W242Q}$)

Supplementary Figure S8: Docking models of (2*S*,3*R*,4*S*)-4-HIL (HILDHE$^{144K/W242Q}$)

Supplementary Figure S9: HPLC chromatogram of stereoisomers of AMKP and 4-HIL

Supplementary Text

Structure determination of the HILDH-NADH, HILDH-NADH-succinate and HILDH$^{E144K/W242Q}$-NADH-succinate complexes

The crystal structures of the HILDH-NADH, HILDH-NADH-succinate and HILDH$^{E144K/W242Q}$-NADH-succinate complexes were determined at 2.20 Å, 2.35 Å and 1.90 Å resolution, respectively. In the structure of the HILDH-NADH complex, there are four HILDH molecules contained per asymmetric unit (Figure S2a). Each molecule is composed of amino-acid residues from Asn4 to Lys246 with an *R*-factor of 16.6% and *R*-free of 20.5%. The structure of HILDH-NADH lacks 16 residues (Ile42–Asp48 and Leu187–Lys195) in chains B, C and D, respectively. Analysis of stereochemical quality by PROCHECK showed that 752 residues fall in the most favored region, 64 residues fall in the additional allowed region and 4 residues fall in the generously allowed region of the Ramachandran plot. The root mean square deviations (RMSD) of four subunits in an asymmetric unit are 0.2-0.3 Å for α-carbons, showing high similarity among subunits.

The structure of HILDH-NADH-succinate contains a homotetramer composed of amino acid residues from Ile6 to Gly245 with an *R*-factor of 21.6% and *R*-free of 25.4% at 2.35 Å. The structure of HILDH-NADH-succinate lacks 6 residues (Glu189-Lys195) in chain A, 26 residues (Ile42–Asp48 and Arg184–Lys202) in chains B and C, respectively, and 29 residues (Leu41–Pro50 and Arg184–Lys202) in chain D. PROCHECK analysis showed that 707, 63 and one residues fall in the most favored, additionally allowed and generously allowed regions of the Ramachandran plot, respectively.

The HILDH$^{E144K/W242Q}$ structures are composed of amino acid residues from K5 to N247 with an

R-factor of 17.4% and *R*-free of 20.1%. PROCHECK analysis shows that 803, 73 and four residues fall in the most favored, the additionally allowed and the generously allowed regions of the Ramachandran plot, respectively. The structure of HILDH$^{E144K/W242Q}$ forms a homotetramer similar to the wild type, with no significant structural difference, at RMSDs of 0.2-0.3 Å.

Supplementary Table S1. Data collection and refinement statistics

	HILDH/NADH	HILDH/NADH/ succinate	HILDH^{E144KW242Q}/ NADH/succinate
Data collection			
X-ray source	PF BL-5A	PF BL-5A	PF BL-17A
Wavelength (Å)	1.0000	1.0000	0.98000
Space group	*R*3	*R*3	*R*3
Unit-cell parameters (Å)	$a = b = 231.5$, $c = 78.7$	$a = b = 232.7$, $c = 80.0$	$a = b = 231.9$, $c = 79.0$
Resolution range (Å)[a]	50.0–2.20 (2.24–2.20)	20.0–2.35 (2.39–2.35)	45.5–1.90 (2.01–1.90)
No. of observed reflections[a]	464,080	373,132	654,834
No. of unique reflections[a]	79,736	66,938	124,974
Average redundancy[a]	5.8 (5.8)	5.6 (5.6)	5.2 (5.2)
Completeness (%)[a]	100 (100)	100.0 (100.0)	99.7 (98.6)
R_{sym} [a,b]	0.081 (0.316)	0.093 (0.687)	0.103 (0.790)
$I/\sigma(I)$[a]	31.6 (6.8)	32.6 (3.2)	16.0 (2.3)
Refinement			
R_{work} / R_{free} (%)[c]	16.6 / 20.5	21.6 / 25.4	17.4 / 20.1
No. of reflections	75,677	63,640	122,874
No. of molecules			
Protein	924	873	972
Ligand	4	4	4
Water	235	90	383
Overall *B* factor (Å^2)	32.9	55.1	25.3
RMSD[d]			
Bond lengths (Å)	0.021	0.016	0.023
Bond angles (°)	2.083	1.934	2.225
Ramachandran plot (%)			
Favored, allowed	91.7, 8.3	91.7, 8.3	91.2, 8.8

[a]Values in parentheses are shown for the highest resolution shell.

[b]$R_{sym} = \Sigma_{hkl} [(\Sigma_i |I_i - \langle I \rangle|) / \Sigma_i |I_i|]$, where I_i is the *i*th intensity measurement of reflection *hkl*, including symmetry-related reflections, and $\langle I \rangle$ is its average.

[c]R_{free} was calculated using 5% of the reflections, which were omitted from the refinement.

[d]RMSD, root-mean-square deviation

Supplementary Table S2. Hydrogen bonds and salt bridges between HILDH and succinate

Interaction		Chain A (Å)	Chain B (Å)
HILDH	succinate		
R88N	O2	2.79	-
S137OH	O3	2.85	2.54
R147N	O1	3.93	3.81
R147N	O2	2.79	3.68
Y150OH	O3	2.90	2.87

Supplementary Table S3. 4-HIL stereoisomers produced by the wild-type and mutant HILDH

4-HIL		Wild-type		R88A		R147A		Y191A	
		Yield (μg/ml)[a]	Production ratio (%)	Yield (μg/ml)[a]	Production ratio (%)	Yield (μg/ml)[a]	Production ratio (%)	Yield (μg/ml)[a]	Production ratio (%)
Total		734.9±76.9	-	180.6±26.0	-	114.3±4.5	-	162.2±3.4	-
4*S* form	2*S*,3*S*,4*S*	92.2± 7.0	12.5	ND[b]	ND[b]	17.5±1.4	15.3	16.1±0.3	9.9
	2*S*,3*R*,4*S*	95.2±10.3	13.0	153.0±23.7	84.7	0.7±0.0	0.6	37.7±0.8	23.2
	2*R*,3*R*,4*S*	138.1±23.7	18.8	3.9± 0.2	2.2	23.4±0.7	20.5	24.7±0.6	15.2
	2*R*,3*S*,4*S*	117.3±10.2	16.0	ND[b]	ND[b]	18.2±0.5	16.0	1.3±0.1	0.8
4*R* form	2*S*,3*S*,4*R*	95.3±16.4	13.0	23.7± 2.1	13.1	19.0±0.6	16.6	73.3±1.5	45.2
	2*S*,3*R*,4*R*	104.5±10.5	14.2	ND[b]	ND[b]	18.5±0.5	16.2	6.0±0.1	3.7
	2*R*,3*S*,4*R*	83.1± 8.8	11.3	ND[b]	ND[b]	10.0±0.5	8.7	1.7±0.1	1.0
	2*R*,3*R*,4*R*	9.2± 4.6	1.3	ND[a]	ND[b]	6.9±0.6	6.1	1.4±0.1	0.9

[a]Mean±standard error (*n*=3)

[b]ND means not detected.

Supplementary Table S4. 4-HIL stereoisomers produced by the R88 mutants

4-HIL	4*S* form (μg/ml)[a]					4*R* form (μg/ml)[a]				
	Yield (μg/ml)[a]				Production ratio (%)	Yield (μg/ml)[a]				Production ratio (%)
	2*S*,3*S*,4*S*	2*S*,3*R*,4*S*	2*R*,3*R*,4*S*	2*R*,3*S*,4*S*	-	2*S*,3*S*,4*R*	2*S*,3*R*,4*R*	2*R*,3*S*,4*R*	2*R*,3*R*,4*R*	-
Wild-type	92.2± 7.0	95.2± 10.3	138.1±23.7	117.3±10.2	60.3	95.3± 16.4	104.5±10.5	83.1±8.8	9.2±4.6	39.7
R88D	ND[b]	147.6± 16.3	ND[b]	ND[b]	94.2	9.1± 2.2	ND[b]	ND[b]	ND[b]	5.8
R88C	ND[b]	277.5± 24.3	ND[b]	ND[b]	88.3	36.7± 4.9	ND[b]	ND[b]	ND[b]	11.7
R88F	ND[b]	375.6±161.3	ND[b]	ND[b]	84.7	67.6± 29.4	ND[b]	ND[b]	ND[b]	15.3
R88G	ND[b]	220.7± 48.1	ND[b]	ND[b]	80.4	53.9± 22.3	ND[b]	ND[b]	ND[b]	19.6
R88A	ND[b]	153.0± 23.7	3.9± 0.2	ND[b]	86.9	23.7± 2.1	ND[b]	ND[b]	ND[b]	13.1
R88E	ND[b]	178.9± 4.8	9.8± 4.1	ND[b]	79.2	49.6± 24.2	ND[b]	ND[b]	ND[b]	20.8
R88H	ND[b]	130.9± 22.0	30.0± 5.6	ND[b]	51.0	154.7± 22.0	ND[b]	ND[b]	ND[b]	49.0
R88T	ND[b]	132.2± 21.6	49.9±15.7	ND[b]	46.0	203.8± 41.3	9.7± 1.0	ND[b]	ND[b]	54.0
R88Q	ND[b]	171.7± 14.8	40.6± 1.9	ND[b]	45.5	228.6± 6.8	26.0± 0.8	ND[b]	ND[b]	54.5
R88S	ND[b]	147.9± 28.3	50.5±11.7	ND[b]	43.1	252.7± 57.9	8.7± 4.5	ND[b]	ND[b]	56.9
R88K	ND[b]	182.8± 15.2	110.8±28.1	ND[b]	39.4	398.2±108.3	28.1± 4.0	26.1±3.0	ND[b]	60.6
R88L	13.3±13.3	167.0± 49.3	27.4±25.8	5.6± 5.6	72.4	75.5± 47.5	5.6± 5.6	ND[b]	ND[b]	27.6
R88N	9.4± 4.9	79.0± 9.9	143.2±16.8	22.6± 2.9	60.8	148.5± 24.8	12.0± 1.3	3.1±1.6	ND[b]	39.2
R88I	48.9± 7.7	120.2± 24.2	113.1±18.4	17.0± 2.8	57.2	197.5± 26.5	21.9± 3.3	4.8±2.4	ND[b]	42.8
R88V	28.6± 1.7	91.0± 9.0	82.5± 4.2	13.1± 4.3	48.6	181.4± 9.3	28.0± 1.3	18.5±0.8	ND[b]	51.4
R88P	ND[b]	ND[b]	ND[b]	ND[b]	-	ND[b]	ND[b]	ND[b]	ND[b]	-
R88M	ND[b]	ND[b]	ND[b]	ND[b]	-	ND[b]	ND[b]	ND[b]	ND[b]	-
R88Y	ND[b]	ND[b]	ND[b]	ND[b]	-	ND[b]	ND[b]	ND[b]	ND[b]	-
R88W	ND[b]	ND[b]	ND[b]	ND[b]	-	ND[b]	ND[b]	ND[b]	ND[b]	-

[a]Mean±standard error (*n*=3)

[b]ND means not detected.

Supplementary Table S5. 4-HIL stereoisomers produced by the W242 mutants.

	(2*S*,3*R*,4*S*)-4-HIL (μg/ml)[a]	(2*S*,3*S*,4*R*)-4-HIL (μg/ml)[a]	Optical purity (% de)
Wild-type	95.2± 10.3	639.7± 67.1[b]	-
W242E	302.5± 42.9	21.9± 5.5	86.5
W242K	296.5± 41.5	28.4± 2.8	82.5
W242N	440.5±132.6	64.2± 16.5	74.6
W242Q	152.4± 2.2	73.9± 1.0	34.7
W242R	316.4± 57.6	380.0±123.0	-

[a]Mean±standard error (*n*=3)

[b]Calculated from the total products of 4-HIL stereoisomers except 2*S*,3*R*,4*S* in the wild-type.

Supplementary Table S6. 4-HIL stereoisomers produced by the R88/W242 double mutants

		(2*S*,3*R*,4*S*)-4-HIL (μg/ml)[a]	(2*S*,3*S*,4*R*)-4-HIL (μg/ml)[a]	Optical purity (% de)
Wild-type		95.2±10.3	639.7±67.1[b]	-
R88C	W242N	172.7±11.7	8.3± 0.6	90.8
	W242E	138.4± 9.8	10.4± 0.7	86.1
	W242K	177.7±13.6	18.6± 3.3	80.1
	W242Q	188.2±31.7	39.6± 9.4	65.2
R88G	W242R	72.8±22.8	12.0± 3.1	71.6

[a]Mean±standard error (*n*=3)

[b]Calculated from the total products of 4-HIL stereoisomers except 2*S*,3*R*,4*S* in the wild-type.

Supplementary Table S7. 4-HIL stereoisomers produced by the E144/W242 double mutants

	(2*S*,3*R*,4*S*)-4-HIL (μg/ml)[a]	(2*S*,3*S*,4*R*)-4-HIL (μg/ml)[a]	Optical purity (% de)
Wild-type	95.2±10.3	639.7±67.1[b]	-
E144K/W242Q	151.9±43.3	0.7± 0.5	99.1
E144K/W242R	245.1±25.9	4.7± 1.0	96.2
E144K/W242E	155.1±29.3	4.8± 2.1	94.0
E144K/W242N	134.6± 7.9	5.2± 0.3	92.5

[a]Mean±standard error (*n*=3)

[b]Calculated from the total products of 4-HIL stereoisomers except 2*S*,3*R*,4*S* in the wild-type.

Supplementary Table S8. Multiple sequence alignment of HILDH$^{E144K/W242Q}$ and SDR homologues searched by BLAST

Accession No.	Organism	Sequence motif 1	Sequence motif 2	Sequence motif 3	Sequence identity (%)
HILDH$^{E144K/W242Q}$	*Bacillus thuringienis* 2e2	144 KPERAAY	187 LTEEYFSN	239 DGGQTAG	-
1 WP_001208940.1	*Bacillus* group	144 EPERAAY	187 LTEEYFSN	239 DGGWTAG	99
2 CKH52088.1	*Streptococcus pneumoniae*	144 EPERAAY	187 LTEEYFSN	239 DGGWTAG	98
3 EEK63229.1	*Bacillus cereus* 172560W	137 EPERAAY	180 LTEEYFSN	232 DGGWTAG	99
4 WP_002144105.1	*Bacillus cereus* group	144 EPERAAY	187 LTEEYFSD	239 DGGWTAG	85
5 EEL70828.1	*Bacillus cereus* AH603	137 EPERAAY	180 LTEEYFSD	232 DGGWTAG	85
6 WP_034642298.1	*Bacillus manliponensis*	144 EPERAAY	187 LTEEYFSN	239 DGGWTAG	81
7 WP_060699640.1	*Bacillus* sp. NH7I_1	143 EPARAAY	186 LTEEYFQN	238 DGGWTAG	62
8 WP_014481373.1|	*Bacillus subtilis*	143 EPARAAY	186 LTEEYFQN	238 DGGWTAG	60
9 WP_015250878.1	*Bacillus subtilis*	143 EPARAAY	186 LTEEYFQN	238 DGGWTAG	60

Supplementary Table S8 (continued)

Accession No.	Organism	Sequence motif 1	Sequence motif 2	Sequence motif 3	Sequence identity (%)
10 KFF55020.1	*Bacillus subtilis*	143 EPARAAY	186 LTEEYFQN	238 DGGWTAG	60
11 COG30553.1	*Streptococcus pneumoniae*	45 EPERAAY	88 LTEEYFSN	140 DGGWTAG	98
12 CON89042.1	*Streptococcus pneumoniae*	143 EPARAAY	186 LTEEYFQD	238 DGGWTAG	60
13 WP_061668978.1	*Bacillus atrophaeus*	143 EPARAAY	186 LTEEYFQD	238 DGGWTAG	60
14 WP_039075018.1	*Bacillus* sp. MSP13	143 EPARAAY	186 LTEEYFQD	238 DGGWTAG	59
15 WP_010845150.1	*Xenorhabdus nematophila*	138 EPNRTAY	181 LTERYYHD	233 DGGWTIG	41
16 WP_058760486.1	*Pseudomonas psychrotolerans*	142 EPNRTAY	185 LTESYYFD	237 DGGWTAG	44
17 WP_047607576.1	*Rahnella aquatilis*	139 EPDRTAY	182 LTEPYYNN	234 DGGWTIG	43
18 WP_022622559.1	Pantoea ananatis	140 EPDRTAY	183 LTESYYHD	235 DGGWTLG	43
19 XP_015872036.1	*Ziziphus jujuba*	149 APKRAAY	192 MTERYFQD	244 DGGWTIG	38
20 WP_040545660.1	*Pelagibaca bermudensis*	147 SENRAAY	190 MTELYFSD	242 DGGYTAG	40

Supplementary Table S8 (continued)

Accession No.	Organism	Sequence motif 1	Sequence motif 2	Sequence motif 3	Sequence identity (%)
21 CRH31318.1	*Pantoea ananatis*	107 EPDRTAY	150 LKESYYHD	202 DGGWTLG	44
22 WP_063891535.1	*Sinorhizobium* sp. GL28	160 IRNRPAY	203 MTEVYYSD	255 DGGFMAE	35
23 WP_005793336.1	*Acidovorax delafieldii*	149 VPNRLAY	192 MITNTLAD	244 DGGNTAG	31
24 WP_059951669.1	*Burkholderia territorii*	149 IPNRASY	192 MTEVMFQD	244 DGGYAAG	33
25 WP_009604570.1	SAR116 cluster alpha *proteobacterium* HIMB100	145 IPAIPAY	188 MMAGVNAN	240 DGGRIGM	35
26 WP_059497368.1	*Burkholderia cepacia*	149 VPNRLAY	192 MTAVMFQD	244 DGGSTAG	33
27 WP_050523171.1	*Pseudorhodobacter wandonensis*	145 IPSIAAY	188 MMAGVNAN	240 DGGRLGM	36
28 WP_036350917.1	*Microvirga lupini*	144 NPRRNAY	187 MVAELER	509 DGGWTSF	35
29 WP_059623746.1	*Burkholderia cepacia*	149 VPNRLAY	192 MTAVMFED	244 DGGSTAG	33
30 WP_041860985.1	*Candidatus Puniceispirillum marinum*	153 GVDRSVY	196 LTEQTFSR	248 DGGWTAD	34

Supplementary Table S8 (continued)

Accession No.	Organism	Sequence motif 1	Sequence motif 2	Sequence motif 3	Sequence identity (%)
31 ADE38290.1	*Candidatus Puniceispirillum marinum*	154 GVDRSVY	197 LTEQTFSR	249 DGGWTAD	34
35 EKE03785.1	uncultured bacterium	146 EPDTAAY	189 WKKKSVKH	242 DGGMTKK	38
36 WP_041761824.1	*Pseudonocardia dioxanivorans*	144 FADRSAY	187 MTRAYFES	239 DGGWNSA	31

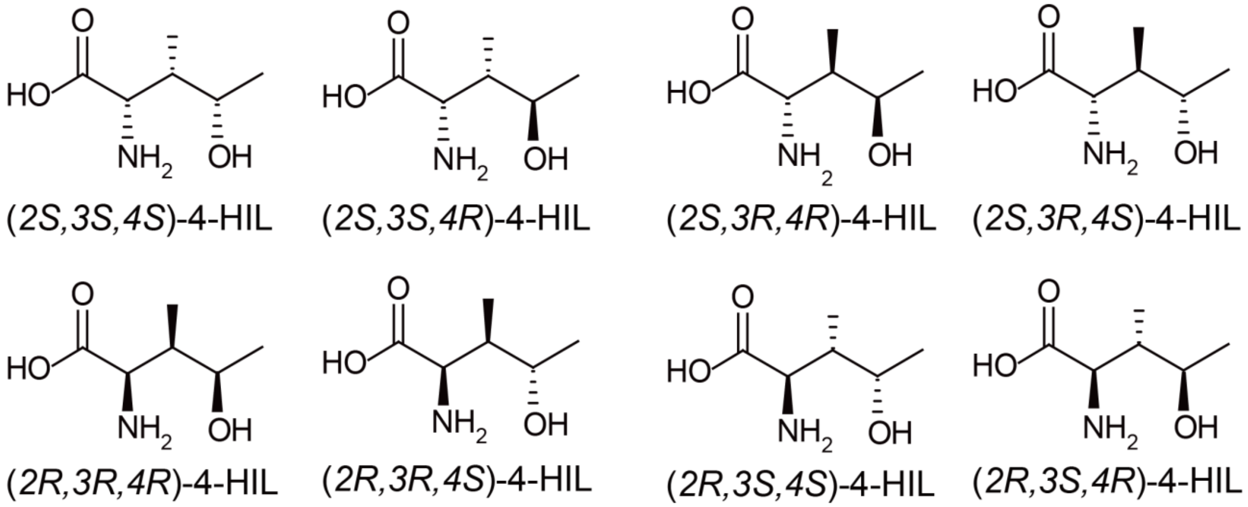

Supplementary Figure S1. Eight stereoisomers of 4-hydroxyisoleucine (4-HIL). 4-HIL has three asymmetric carbons and hence eight 4-HIL stereoisomers exist.

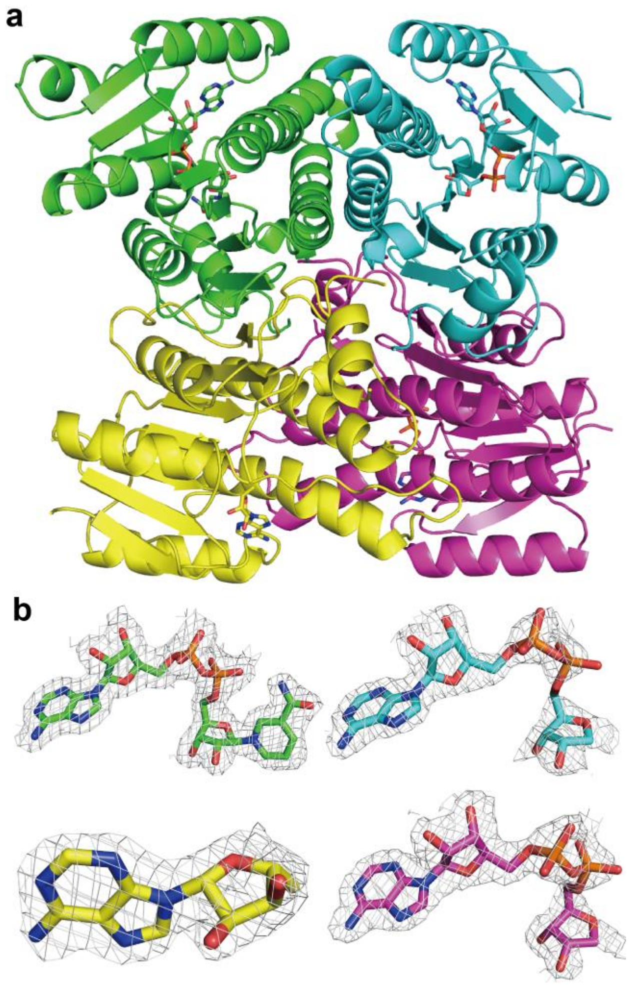

Supplementary Figure S2. Overall structure of HILDH. (**a**) Ribbon representation of the tetrameric state of HILDH-NADH in an asymmetric unit. The four subunits are colored green, cyan, magenta and yellow. The NADH molecules are represented as stick models. (**b**) F_0 - F_c electron density omit maps of NADH contoured at 1.0 σ bound by chains A (green), B (cyan), C (magenta), and D (yellow).

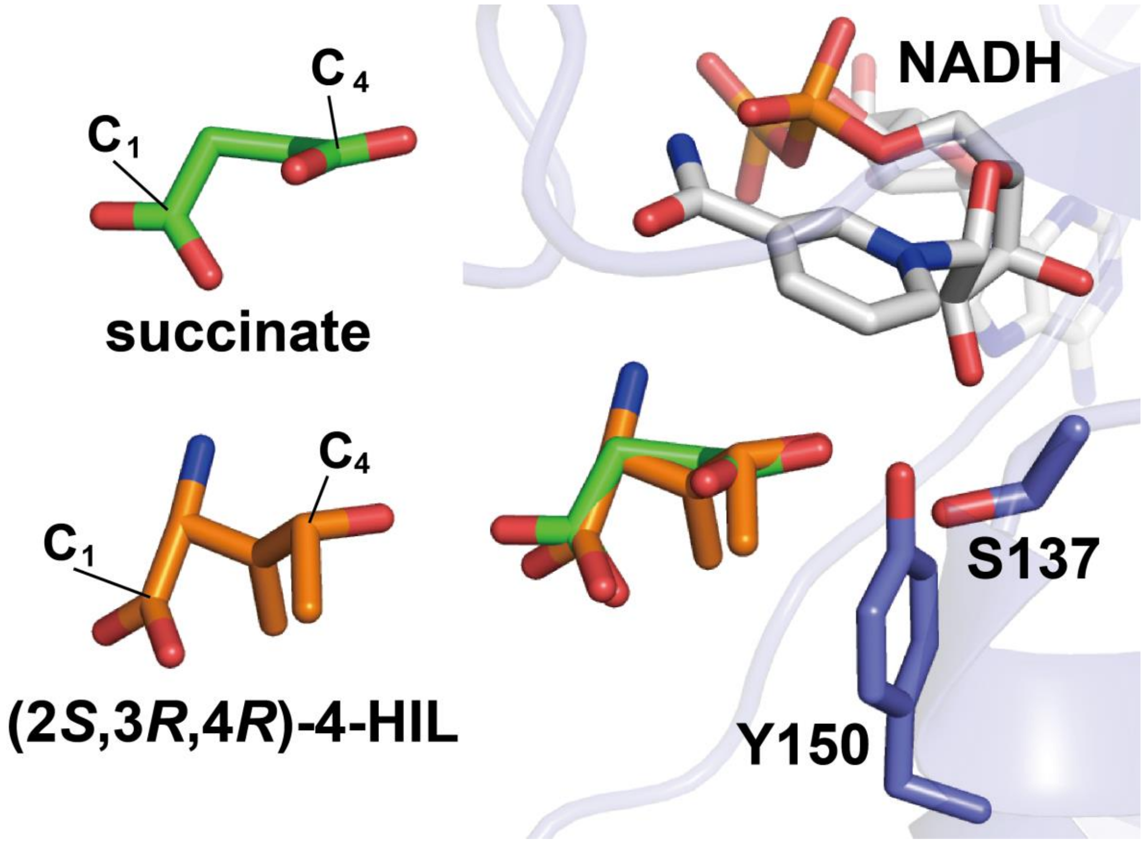

Supplementary Figure S3. Superimposition of (2*S*,3*R*,4*R*)-4-HIL onto succinate. Succinate, (2*S*,3*R*,4*R*)-4-HIL, HILDH and NADH are shown in green, orange, slate and white sticks, respectively.

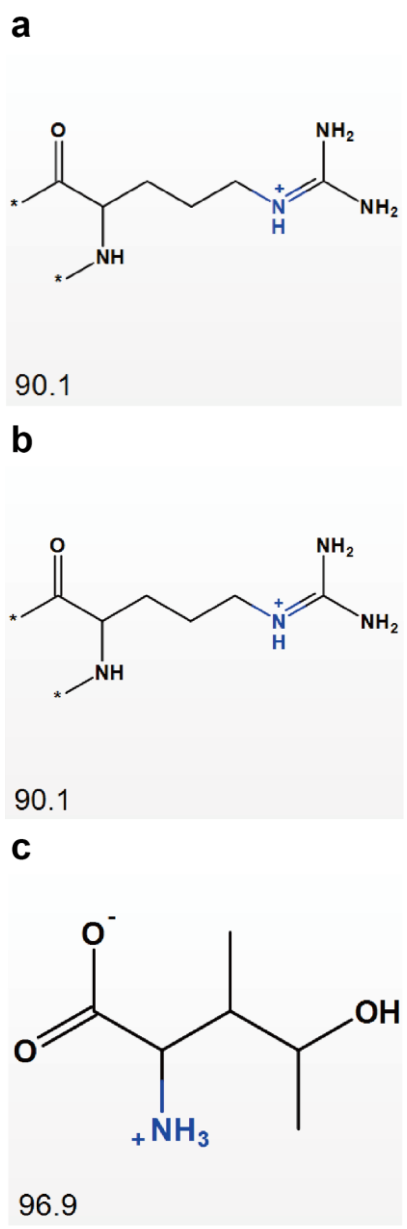

Supplementary Figure S4. Simulated protomer/tautomer states at pH 9.1. Different protomer/tautomer states of R88 (a), R147 (b) and 4-HIL (c) at pH 9.1 were calculated by MOE. The evaluation criteria shown on the lower left of individual figures mean the proportion of the protonation state and its tautomer (%).

	mol	mseq	U_dock	U_total	U_ele	U_vdw	U_ligand	U_strain	U_solv	score	scoreV	scoreX	site	conf
1	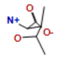	1	-84.4715	-80.1350	-100.4357	5.5216	14.7791	10.4426	0.0000	-18.7262	-19.6271	0.9009	1	3
2	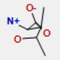	1	-82.4800	-78.1438	-98.2129	5.3354	14.7337	10.3975	0.0000	-18.8530	-19.7490	0.8959	1	4
3	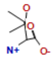	1	-77.6359	-59.6237	-101.3863	8.6365	33.1261	15.1138	0.0000	-18.0065	-18.9660	0.9595	1	5
4	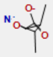	1	-73.7457	-69.4094	-89.5676	9.4860	10.6723	6.3360	0.0000	-19.3594	-20.2367	0.8773	1	2
5	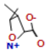	1	-67.4344	-63.0968	-91.9731	6.6686	22.2077	17.8700	0.0000	-16.4091	-17.3297	0.9206	1	3
6	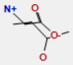	1	-66.7215	-56.2727	-89.8924	3.1549	30.4648	20.0160	0.0000	-21.2166	-21.8821	0.6655	1	4
7	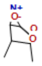	1	-64.1244	-62.7589	-80.3137	2.1339	15.4209	14.0555	0.0000	-21.6962	-22.2027	0.5065	1	1
8	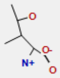	1	-53.9607	-42.2726	-74.3409	8.5003	23.5680	11.8799	0.0000	-19.2114	-20.0961	0.8846	1	3
9	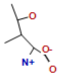	1	-53.8539	-42.1655	-74.3265	8.6159	23.5452	11.8567	0.0000	-19.2023	-20.0885	0.8862	1	3
10	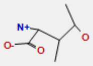	1	-17.0177	1.0080	-20.0690	-2.9131	23.9901	5.9644	0.0000	-20.8829	-21.5004	0.6175	1	5

Supplementary Figure S5. Docking results of (2*S*,3*R*,4*S*)-4-HIL (wild-type HILDH).

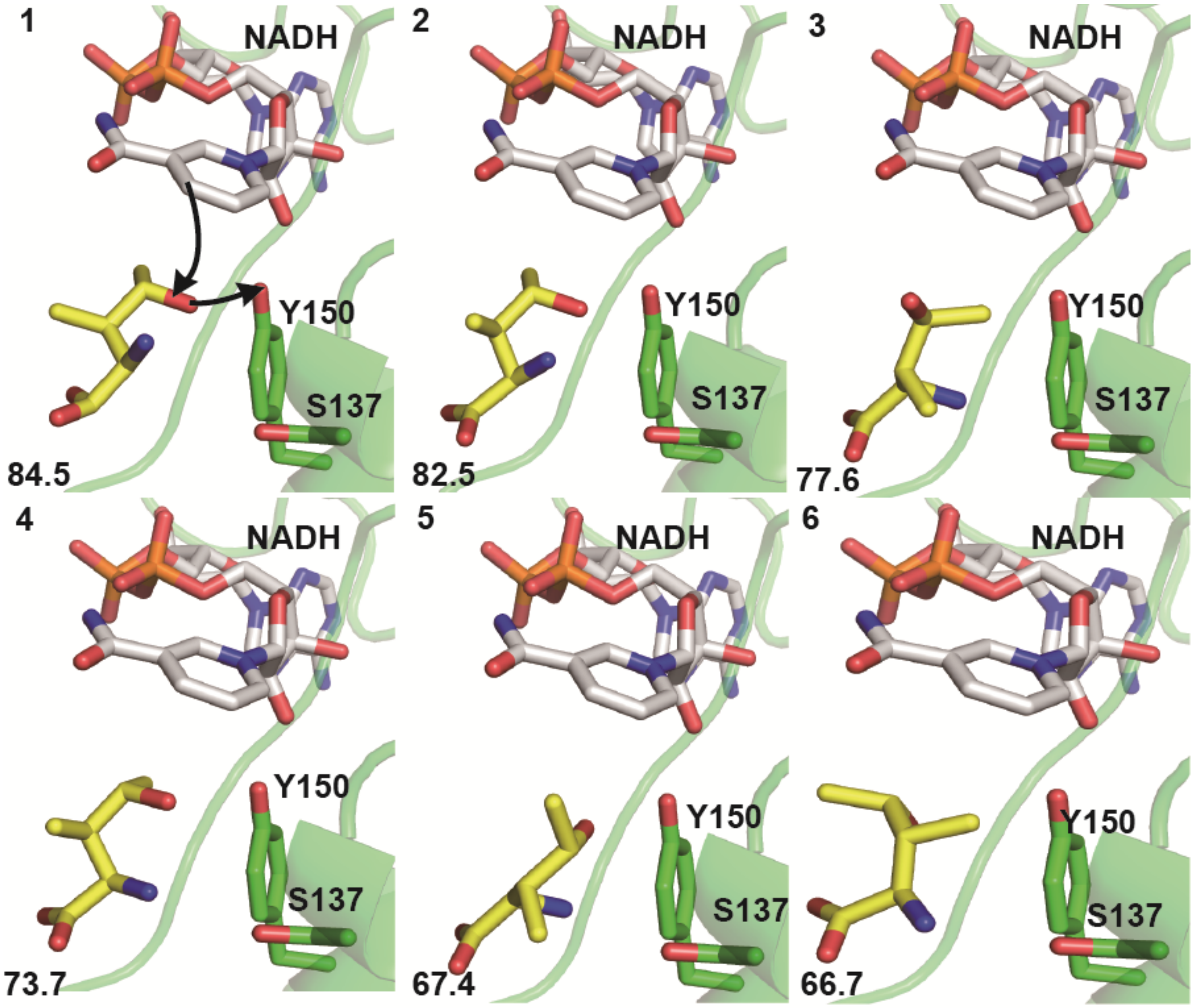

Supplementary Figure S6. Docking models of (2*S*,3*R*,4*S*)-4-HIL (wild-type HILDH). The first six models sorted by the rank of U_dock score (kcal/mol). U_dock scores are shown on the lower left of individual figures. (2*S*,3*R*,4*S*)-4-HIL, HILDH and NADH are shown in yellow, green and white sticks, respectively. The hydrogen transfer direction is shown with an arrow.

	mol	mseq	U_dock	U_total	U_ele	U_vdw	U_ligand	U_strain	U_solv	score	scoreV	scoreX	site	conf
1	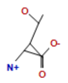	1	-83.5269	-74.3958	-86.0420	-2.2175	13.8637	4.7326	0.0000	-24.4058	-24.8198	0.4140	1	3
2	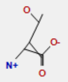	1	-81.5729	-72.4435	-89.7689	-1.6421	18.9675	9.8381	0.0000	-24.3666	-24.7973	0.4307	1	3
3	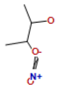	1	-76.0149	-66.8854	-87.4313	-3.8070	24.3529	15.2234	0.0000	-21.9458	-22.5584	0.6126	1	3
4	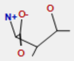	1	-73.6405	-71.6183	-82.0925	-3.8916	14.3658	12.3436	0.0000	-22.2862	-22.6819	0.3956	1	1
5	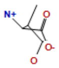	1	-59.6588	-50.5289	-94.4356	16.6000	27.3067	18.1768	0.0000	-20.5126	-21.3242	0.8116	1	2
6	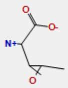	1	-47.5999	-33.8488	-69.6504	4.3749	31.4267	17.6755	0.0000	-23.2235	-23.9570	0.7335	1	3
7	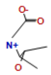	1	-47.2798	-33.5289	-70.1535	12.0470	24.5777	10.8268	0.0000	-21.9173	-22.7468	0.8296	1	2
8	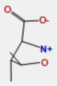	1	-31.8103	-29.7875	-39.1644	-3.3360	12.7129	10.6900	0.0000	-23.8011	-24.2657	0.4646	1	1
9	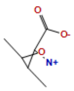	1	-20.8667	-12.9951	-40.2266	-0.0825	27.3140	19.4424	0.0000	-20.6145	-21.0631	0.4487	1	1

Supplementary Figure S7. Docking results of (2*S*,3*R*,4*S*)-4-HIL (HILDH$^{E144K/W242Q}$).

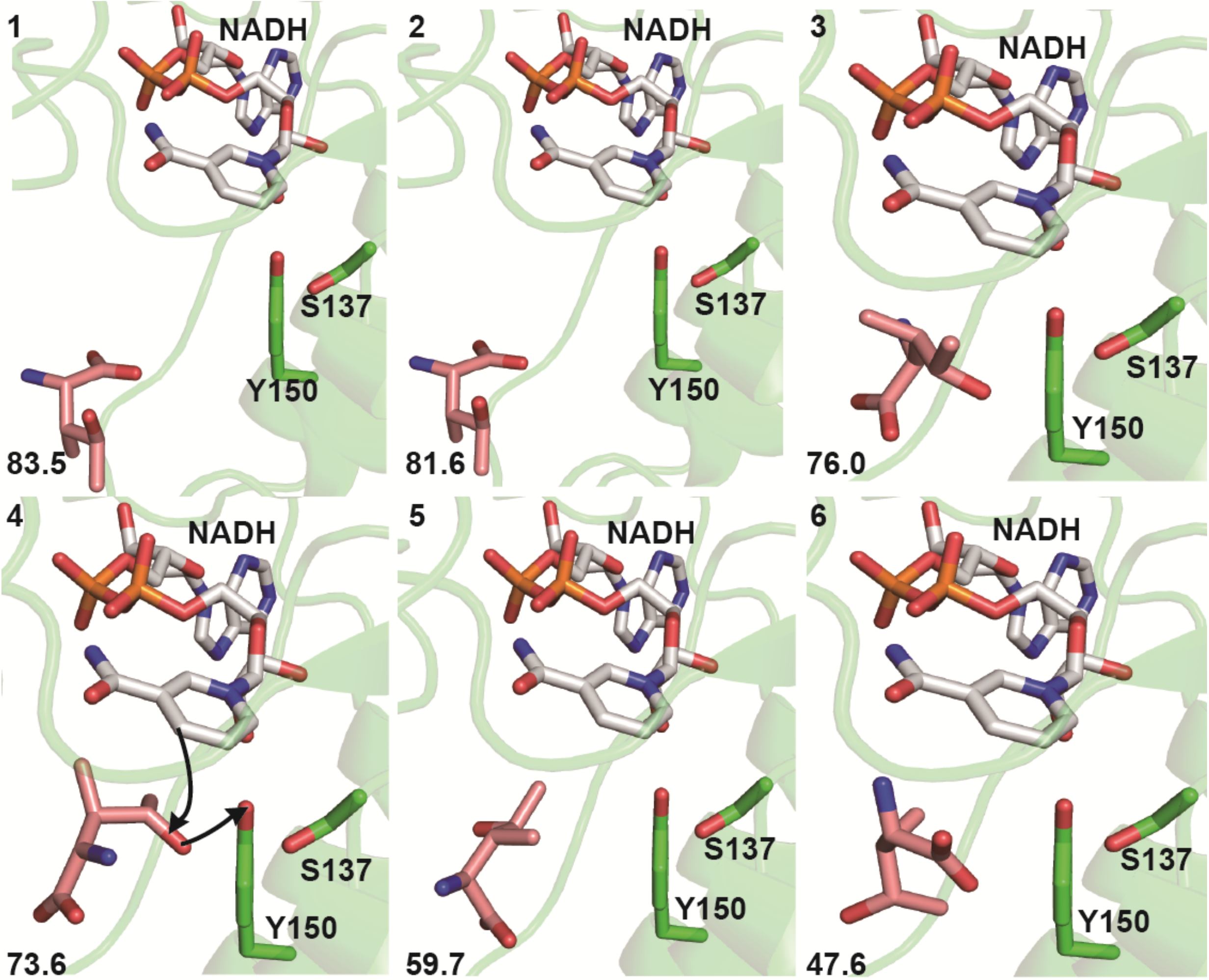

Supplementary Figure S8. Docking models of (2*S*,3*R*,4*S*)-4-HIL (HILDH$^{E144K/W242Q}$). The first six models sorted by the rank of U_dock score (kcal/mol). U_dock scores are shown on the lower left of individual figures. (2*S*,3*R*,4*S*)-4-HIL, HILDH and NADH are shown in pink, green and white sticks, respectively. The hydrogen transfer direction is shown with an arrow.

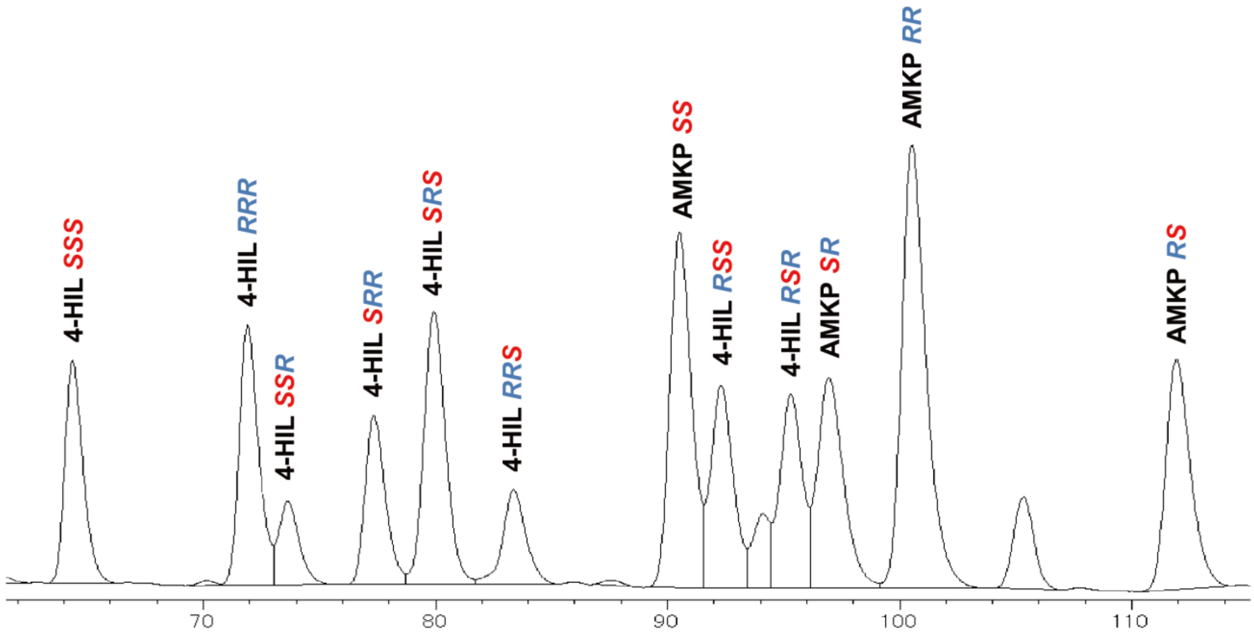

Supplementary Figure S9. HPLC chromatogram of stereoisomers of AMKP and 4-HIL. The mixtures of racemic AMKP and racemic 4-HIL were derivatized with GITC and detected by HPLC.
